# *Cascabel*: A Scalable and Versatile Amplicon Sequence Data Analysis Pipeline Delivering Reproducible and Documented Results

**DOI:** 10.3389/fgene.2020.489357

**Published:** 2020-11-20

**Authors:** Alejandro Abdala Asbun, Marc A. Besseling, Sergio Balzano, Judith D. L. van Bleijswijk, Harry J. Witte, Laura Villanueva, Julia C. Engelmann

**Affiliations:** ^1^Department of Marine Microbiology and Biogeochemistry, NIOZ Royal Netherlands Institute for Sea Research, Texel, Netherlands; ^2^Department of Earth Sciences, Faculty of Geosciences, Utrecht University, Utrecht, Netherlands

**Keywords:** amplicon sequencing, 16S/18S rRNA gene, Illumina, community profiling, microbiome, pipeline, snakemake

## Abstract

Marker gene sequencing of the rRNA operon (16S, 18S, ITS) or cytochrome c oxidase I (CO1) is a popular means to assess microbial communities of the environment, microbiomes associated with plants and animals, as well as communities of multicellular organisms *via* environmental DNA sequencing. Since this technique is based on sequencing a single gene, or even only parts of a single gene rather than the entire genome, the number of reads needed per sample to assess the microbial community structure is lower than that required for metagenome sequencing. This makes marker gene sequencing affordable to nearly any laboratory. Despite the relative ease and cost-efficiency of data generation, analyzing the resulting sequence data requires computational skills that may go beyond the standard repertoire of a current molecular biologist/ecologist. We have developed *Cascabel*, a scalable, flexible, and easy-to-use amplicon sequence data analysis pipeline, which uses Snakemake and a combination of existing and newly developed solutions for its computational steps. *Cascabel* takes the raw data as input and delivers a table of operational taxonomic units (OTUs) or Amplicon Sequence Variants (ASVs) in BIOM and text format and representative sequences. *Cascabel* is a highly versatile software that allows users to customize several steps of the pipeline, such as selecting from a set of OTU clustering methods or performing ASV analysis. In addition, we designed *Cascabel* to run in any linux/unix computing environment from desktop computers to computing servers making use of parallel processing if possible. The analyses and results are fully reproducible and documented in an HTML and optional pdf report. *Cascabel* is freely available at Github: https://github.com/AlejandroAb/CASCABEL.

## 1. Introduction

High-throughput sequencing of an omnipresent marker gene, such as the gene coding for the small subunit of the ribosomal RNA (16S for prokaryotes or 18S for eukaryotes) is a cost-efficient means for community profiling that is affordable for nearly every lab. On current sequencing platforms, up to hundreds of samples can be combined (multiplexed) in a single sequencing run, decreasing the sequencing costs per sample tremendously, and generating massive amounts of data. Not surprisingly, community compositions based on DNA analyses have been generated from most of the habitats on earth, including the human body (Human Microbiome Project Consortium, 2012), the open ocean (Sunagawa et al., 2015), deep sea (Sogin et al., 2006), and intracellular symbionts (Balzano et al., 2015). Moreover, sequencing a marker gene like cytochrome c oxidase I (CO1) or mitochondrial 12S in environmental DNA also allows to track larger multicellular organisms, for example fish in the sea (Hänfling et al., 2016; van Bleijswijk et al., 2020). Amplicon sequencing can also be used to investigate active microbial communities based on ribosomal RNA abundance instead of the rRNA gene locus (Massana et al., 2015; Forster et al., 2016). Typically, a short fragment of 100–600 nucleotides of the marker gene is amplified by PCR from the DNA extract or cDNA generated from the rRNA extract of the community, and then sequenced by high throughput sequencing. During sequence analysis, sequences are often grouped in Operational Taxonomic Units (OTUs) following one of two main strategies: *de novo* or *closed-reference* OTU picking (Westcott and Schloss, 2015). With closed-reference OTU picking, sequences are assigned to a sequence from a reference database given an identity threshold. Sequences which are not similar enough to any sequence in the database are excluded from downstream analyses.

*De novo* OTU picking clusters reads sharing a predefined sequence identity, commonly 97%, yielding approximately species resolution considering the entire 16S rRNA gene (about 1,500 nt) (Stackebrandt and Goebel, 1994). Although widely used, its application to short read sequencing data has been criticized because the individual variable regions of the 16S rRNA gene have quite different taxonomic resolution for different groups of organisms, such that it is impossible to find a general cutoff of sequence identity which would reliably distinguish species (Johnson et al., 2019). For eukaryotes, the situation is similar with respect to the taxonomic resolution at a given sequence identity threshold of the 18S gene, as this can vary even within the same taxonomic group. For example, it has been shown that within diatoms, *Nitzschia* and *Thalassiosira* species can be easily separated based on the diversity of their 18S rRNA gene (Hoppenrath et al., 2007; Rimet et al., 2011) whereas distinct *Pseudo-nitzschia* and *Chaetoceros* species share identical 18S rRNA gene sequences (Amato et al., 2007; Balzano et al., 2017).

In response to the criticism of OTUs, alternative methods have been developed which model sequencing errors to estimate the true biological sequence. DADA2 (Callahan et al., 2016) and deblur (Amir et al., 2017) cluster reads such that the clusters are consistent with the error model, while Minimum entropy Decomposition (MED) (Eren et al., 2015) and Swarm (Mahé et al., 2015) assume that sequence errors occur randomly and they use this assumption and abundance information of unique sequences to cluster them into supposedly biological entities. To set them apart from OTUs, the term “Amplicon Sequence Variant (ASV)” has been coined for results from denoising algorithms, such as DADA2 and Deblur, while MED uses “oligotypes” and Swarm “swarms” for their clusters. All of these approaches do not require setting a sequence identity threshold, and the resolution is determined by the data, which seems to better reflect the true state of nature (Caruso et al., 2019). However, OTU methods are still widely being used and might deliver useful insights for applications where lower taxonomic resolution is sufficient.

While the experimental part of community profiling studies is fairly simple (DNA extraction, PCR), the current bottleneck is the computational analysis of the (potentially massive) sequence data. For scientists with little background in bioinformatics, the amount of data and complexity of data analysis can be overwhelming. Popular software solutions for the individual steps from raw sequence data to an OTU or ASV table, e.g., QIIME (Caporaso et al., 2010b), mothur (Schloss et al., 2009), and DADA2 (Callahan et al., 2016), are not necessarily straightforward to use. The software package mothur (Schloss et al., 2009), which comes with its own computational environment, and the QIIME framework (Caporaso et al., 2010b) both require the ability to work on the command line. Analyzing multiple sequencing libraries quickly becomes tedious for users not proficient in implementing bash (or any other programming language) scripts which chain the individual steps and allow parallel processing. The ASV analysis tool DADA2 (Callahan et al., 2016) comes as an R package, which also requires some scripting skills. While web servers for microbial community data analysis like SILVAngs (Quast et al., 2013) and MG-RAST (Glass et al., 2010), NGTax2 (Poncheewin et al., 2019) and SLIM (Dufresne et al., 2019) are easy to use, they are inherently inflexible and also limited in throughput. QIIME2 (Bolyen et al., 2018) has command line and graphical user interface (GUI) modes of operation and offers even a larger choice of algorithms for data analysis than the original QIIME, including statistical analyses of the resulting community profiles. The GUI has limited functionality though and might not be a convenient solution for analyzing many samples. The same holds for recently developed GUIs like BTW (Morais et al., 2018) and SEED2 (Vetrovský et al., 2018) which run under Microsoft Windows, and PipeCraft (Anslan et al., 2017) which provides a GUI running on Linux systems. For example, none of these three provide an ASV analysis method. More recently developed pipelines which run on the command line focus on usability with minimal bioinformatic skills, but allowing higher throughput than a webserver. These recent pipelines frequently chain existing tools to make them more accessible, but often at the cost of flexibility due to fixed parameter settings, e.g. BMPOS (Pylro et al., 2016), BTW (Morais et al., 2018), and MetaAmp (Dong et al., 2017), or fixed reference databases, like PEMA (Zafeiropoulos et al., 2020). Others miss essential functionality which requires additional tools to make them useful, e.g., PEMA (Zafeiropoulos et al., 2020), and iMAP (Buza et al., 2019) do not provide demultiplexing of sequence libraries.

None of the easy-to-use tools cited above allow the analysis of sequence read pairs which are not overlapping (DADA2 supports non-overlapping reads, but requires R skills). Although most often amplicons are designed and sequenced such that forward and reverse reads overlap and can be merged into one continuous sequence, for example the primer pair 515F (GTGCCAGCMGCCGCGGTAA), 926R (CCGYCAATTYMTTTRAGTTT) amplifies bacterial and archaeal 16S as well as eukaryotic 18S regions (Needham and Fuhrman, 2016). This makes it a cost-efficient approach if both prokaryotic and eukaryotic communities are of interest. With marine environmental samples, the primers produce an amplicon of on average 411 nucleotides originating from prokaryotic sequences and an amplicon of on average 585 nucleotides derived from eukaryotic sequences. Forward and reverse reads from the longer eukaryotic amplicon typically do not overlap sufficiently (current maximum read length of an Illumina MiSeq is 2 × 300 nucleotides) to merge both reads, especially when amplicon length varies between species, and low quality and adapter sequences are trimmed from the reads. The MeFit pipeline (Parikh et al., 2016) can merge forward and reverse reads with N characters, but it is merely a merging and filtering tool, not a complete amplicon data analysis pipeline. A complete workflow to carry on with this kind of analysis is currently not available, to the best of our knowledge. *Cascabel* allows to “stitch” together the forward and reverse non- or not sufficiently overlapping read pairs with any character and continues with the analysis. In existing pipelines, these sequences would be discarded.

Moreover, most of the existing tools do not have documentation functions to guarantee reproducibility and facilitate communicating which software tools, their versions and parameter settings were used. We could identify only one tool, iMAP (Buza et al., 2019), developed at the same time as *Cascabel*, which can generate a report of the analysis. However, iMAP requires editing and adapting bash scripts before it can be run and is therefore not very user-friendly.

Therefore, we anticipated a need for a pipeline which combines the flexibility and scalability provided by using bioinformatic tools on the command line with the ease of using interactive web servers for analyzing and interpreting amplicon sequencing data.

Moreover, issues with reproducibility of research findings have made data provenance an important aspect of data analysis and scientific journals start to require documentation of data provenance for submitted manuscripts (www.nature.com, 2019). Not all pipelines are transparent enough to trace back all the exact steps taken by underlying software and their versions used within the pipeline. With interactive webservers it is often impossible to reproduce an analysis because once the webserver is updated, previous versions are no longer accessible, or, even worse, the webserver changes without the user noticing. Even if versions are documented and previous versions are available, it is the responsibility of the user to actively record all parameter settings in an unambiguous way, which is an error-prone endeavor. Also, this information cannot be recovered at a later time point, and wrong documentations are likely to go unnoticed.

One of the main strengths of *Cascabel* is that all analyses (runs) performed are completely documented and reproducible. All scripts of *Cascabel* are located within the project folder, and together with the Snakemake and configuration file, every run of the pipeline is completely reproducible at any time. All software versions used are documented in the run reports (in HTML and pdf format). The code of all *Cascabel* scripts is open source, although we use some third-party modules which are not open source, e.g., UCLUST, USEARCH (Edgar, 2010).

We here provide *Cascabel*, a Snakemake (Köster and Rahmann, 2012) pipeline for the analysis of community marker gene sequence data which is easy to use for people with little bioinformatics background, and both flexible and powerful enough to be attractive for people with bioinformatics training. *Cascabel* supports large sample and sequencing library throughput as well as parallel computing on personal computers and computing servers. Moreover, the results are summarized in an HTML and pdf report, and all input and output files, tools, parameters and their versions are documented, rendering the analyses fully reproducible.

## 2. Implementation

Our pipeline makes use of the workflow management engine Snakemake (Köster and Rahmann, 2012), which scales from personal workstations to computer clusters. *Cascabel* consists of a set of “rules” which specify the input, the action to perform on the input (executed by a bash/python/R/java script), and the output. The user defines *via* a configuration file (called “config file” from now on) in yaml format, how these “rules” are chained to perform amplicon sequence data analysis from the raw data to the final OTU or ASV table. *Cascabel* saves the OTU or ASV table in BIOM and text format to allow further analysis and interpretation with statistical or visualization tools. For most of the rules, *Cascabel* provides several alternative algorithms or tools and allows passing arguments *via* the config file to the algorithm being used. In addition, rules can be skipped, and the pipeline can be entered and exited at every step. This makes *Cascabel* very flexible and highly customizable. Moreover, the pipeline is easily extendable and amendable to personal needs, allowing for example the analysis of any marker gene sequence data.

In addition to securing data provenance, *Cascabel* has a suite of rules (modules) which are not readily available or straightforward for a non-bioinformatician to implement with existing amplicon sequence data analysis tools. The first one is a custom dereplication rule for very large data sets based on VSEARCH (Rognes et al., 2016), which, depending on the duplication level of the sequence data, can up to double the number of reads which can be dereplicated on a given system. Second, *Cascabel* supports the analysis of non-overlapping read pairs arising from long amplicons. *Cascabel* can “stitch” these reads together with any desired sequence of characters, e.g., one or several N and then proceeds with OTU or ASV analysis. We recommend using RDP for taxonomic classification as this k-mer based method is not affected by additional N characters.

Furthermore, *Cascabel* can generate data files for submission to a public sequence read archive. Demultiplexed fastq files can be generated with barcodes, or barcodes and primers/adapters removed, ready for submission. Another unique feature of *Cascabel* is that the user can determine the level of interaction with the pipeline. In interactive mode, the user is informed about the results of individual rules and can amend parameters during runtime, while in non-interactive mode the pipeline will proceed according to the parameter settings in the config file. We outline the individual steps performed by *Cascabel* below.

**Figure 1:**
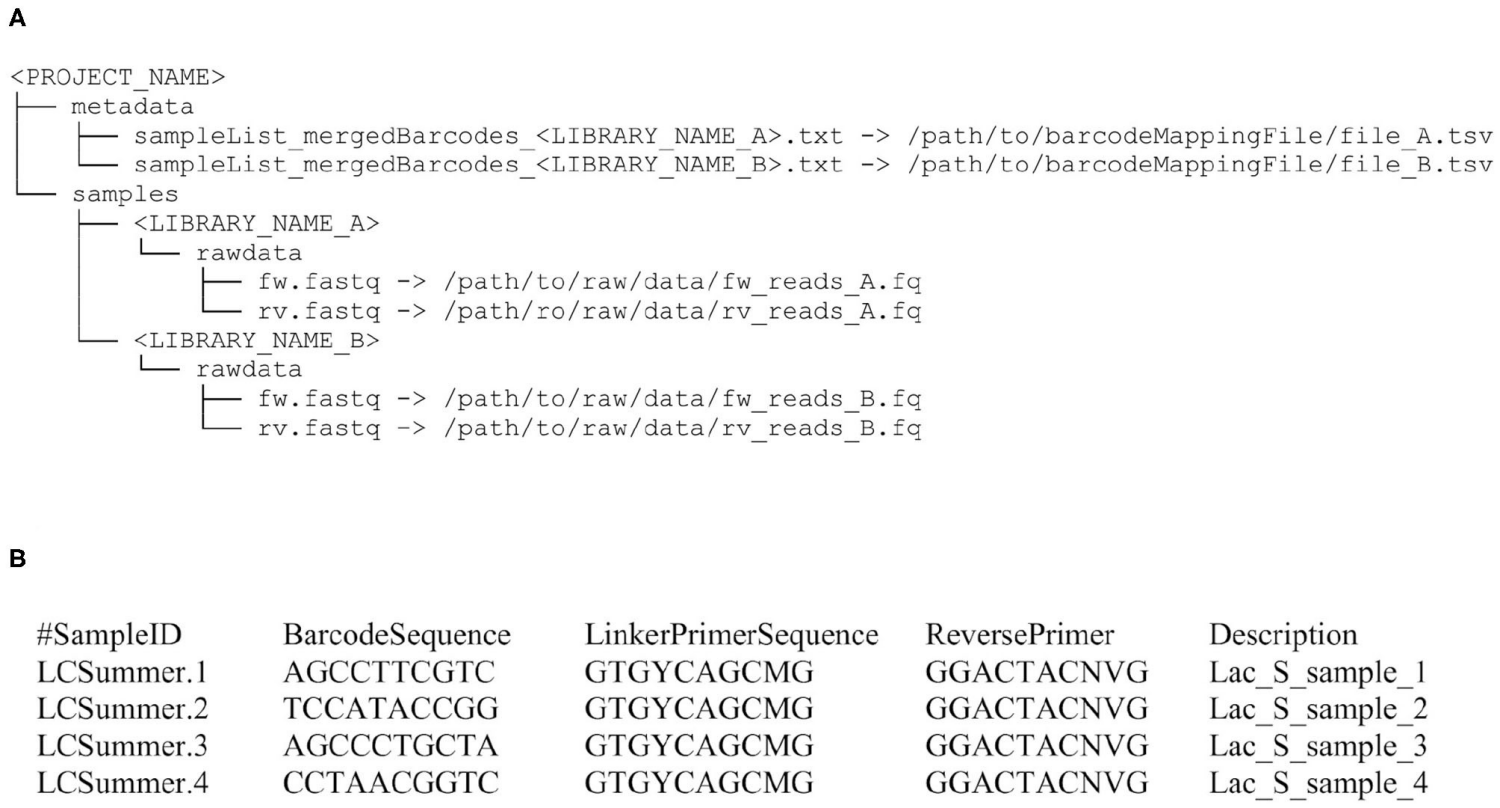
Input file structure for *Cascabel*. **(A)** This input file structure is generated from the file paths provided in the config file when the dataset consists of a single sequencing library. For multiple libraries, it is created from a text file specifying the individual libraries or by the helper script initSample.sh. **(B)** Example of a barcode mapping file for four samples. Barcode and primer sequences are listed in 5′-3′ direction and have been abbreviated.

Running *Cascabel* requires the raw fastq sequence data files, a mapping file indicating which sample carries which barcode if the data should be demultiplexed, and optionally sample metadata (e.g., geographic coordinates of the sampling stations, physical, chemical, or biological properties), and the config file specifying the tools and parameters used for running the pipeline. When working with one sequencing library, users can pass file paths to the raw data and metadata directly in the config file. When working with multiple libraries, users can choose between listing the input file names in a text file and referring to this file in the config file, or using the helper script *initSample.sh*. This script is run for each library to initialize the folder structure expected by *Cascabel*. With all three options, the folder structure will look like the one illustrated in Figure 1A.

**Figure 2:**
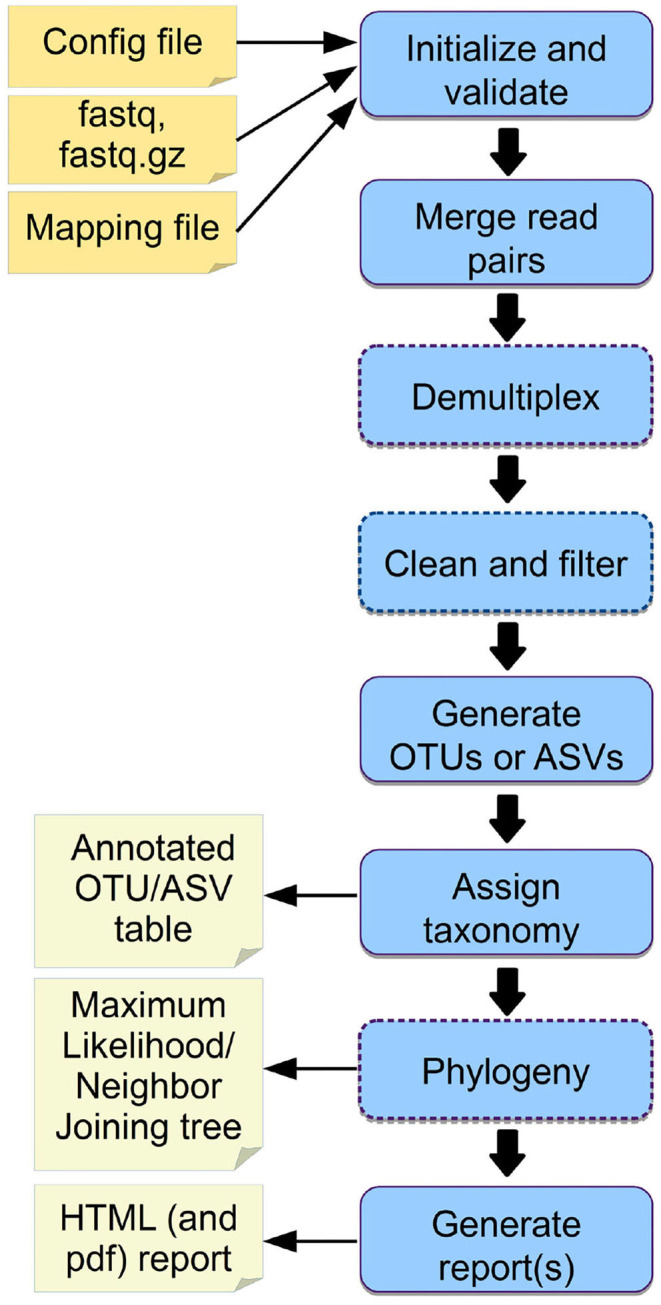
Overview of *Cascabel*. The workflow indicates input files (config file, sequence data in fastq format, barcode mapping file), mandatory and optional steps of the pipeline (blue boxes) as well as the main output files. The boxes of optional steps have dashed borders. “Clean and filter” refers to removing primers/adapters and chimeras. Table 1 shows a detailed summary of the steps, available tools and output files.

We provide example config files with default parameters for double- and single barcoded paired-end reads for OTU and ASV analysis on the github page of *Cascabel* (https://github.com/AlejandroAb/CASCABEL). However, we strongly advise to make informed choices about parameter settings matching the individual needs of the experiment and data set. With the files in place, *Cascabel* is started with a one-line command on the terminal. Snakemake takes care of executing the rules in a computationally efficient manner, making optimal use of available resources, e.g., distributing jobs over several nodes. Figure 2 provides an overview of the workflow of *Cascabel*. In Table 1 we summarize the options and methods provided for the individual steps of the analysis performed by *Cascabel*.

**Table 1:** Outline of the steps performed by *Cascabel*. “Script(s)” refers to *Cascabel* scripts in bash, java or R.

**Step**	**Tools/Algorithms**	**Output**
Initialize structure	Script	Project folder and file structure
Quality Control	FastQC (Andrews, 2010)	FastQC report
Merge reads	PEAR (Zhang et al., 2014)	Merged (assembled) sequences
Demultiplex	QIIME (Caporaso et al., 2010b), scripts	Sequences assigned to samples in one file and per sample
Align vs. reference	Mothur (Schloss et al., 2009)	Aligned sequences
Remove chimeras	usearch61 (Edgar, 2010), Uchime_denovo and uchime_ref (VSEARCH) (Rognes et al., 2016)	Chimera-free sequences
Remove adapters	Cutadapt (Martin, 2011)	Adapter-free sequences
Size filter	Script	Filtered sequences
Dereplicate	VSEARCH	Dereplicated sequences
Generate OTUs	Mothur (Schloss et al., 2009), prefix/suffix (Caporaso et al., 2010b), CD-HIT (Li and Godzik, 2006), SUMACLUST (Kopylova et al., 2016), Swarm (Mahé et al., 2015), UCLUST (Edgar, 2010), trie (Caporaso et al., 2010b) sortmerna (Kopylova et al., 2012)	OTU table
Pick representatives (OTUs)	Random, longest, most_abundant, first	Fasta file with representative sequences
Generate ASVs	DADA2 (Callahan et al., 2016)	ASV table
Assign taxonomy OTUs	QIIME [BLAST (Altschul et al., 1990), UCLUST, RDP (Wang et al., 2007)], blastn (BLAST+) (Camacho et al., 2009), VSEARCH	Taxonomic assignments for each OTU
Assign taxonomy ASVs	RDP	Taxonomic assignments for each ASV
Generate OTU table	QIIME, scripts	Annotated OTU table
Generate ASV table	DADA2	Annotated ASV table
Alignment	Pynast (Caporaso et al., 2010a), mafft (Katoh and Standley, 2013), infernal (Nawrocki and Eddy, 2013), clustalw (Larkin et al., 2007), muscle (Edgar, 2004)	Multiple sequence alignment
Make tree	Muscle, clustalw, raxml (Stamatakis, 2006), fasttree (Price et al., 2009)	Phylogenetic tree
Report	Scripts, Krona (Ondov et al., 2011)	HTML, pdf report, Krona charts

*Cascabel* has an interactive and a non-interactive mode. In interactive mode, several modules have a check-point which needs to be passed to continue with the analysis. If the check fails (e.g., if too many FastQC (Andrews, 2010) quality modules failed or the number of sequences assigned to sample barcodes is too low), the pipeline stops and the user has to decide to continue, change parameters and continue, or exit the pipeline. If parameters were changed interactively, the new ones are documented in the reports. The interactive mode is useful in the explorative data analysis stage, while the non-interactive mode is suitable for running large batches of data and evaluating the results later.

The first step of *Cascabel* consists of checking the validity of the input files including the barcode mapping file and the config file. *Cascabel* supports single-end as well as paired-end sequence data as input from one or multiple samples per input file. Barcodes for demultiplexing samples can be situated at the beginning of one or both of the reads. The barcode sequences are read from the barcode mapping file, which is exemplified in Figure 1B. Supplementary Datasheets 1, 2 contain sample config files, which were used to generate the reports provided in Supplementary Datasheets 3–5. After having validated the input files, *Cascabel* proceeds with analyzing sequence data quality with FastQC (Andrews, 2010). In interactive mode, *Cascabel* will stop if more than a specified number of quality check modules failed. Next, read pairs are assembled with PEAR (Zhang et al., 2014) and the quality of the assembled reads is again assessed with FastQC. *Cascabel* also offers an “unpaired” workflow for paired-end sequence data with non-overlapping reads. For this kind of data, *Cascabel* merges the forward and reverse read with an “N” or any other character, and assigns taxonomy using the RDP classifier, which, due to using a k-mer approach, is not impacted by this procedure (Jeraldo et al., 2014).

**Figure 3:**
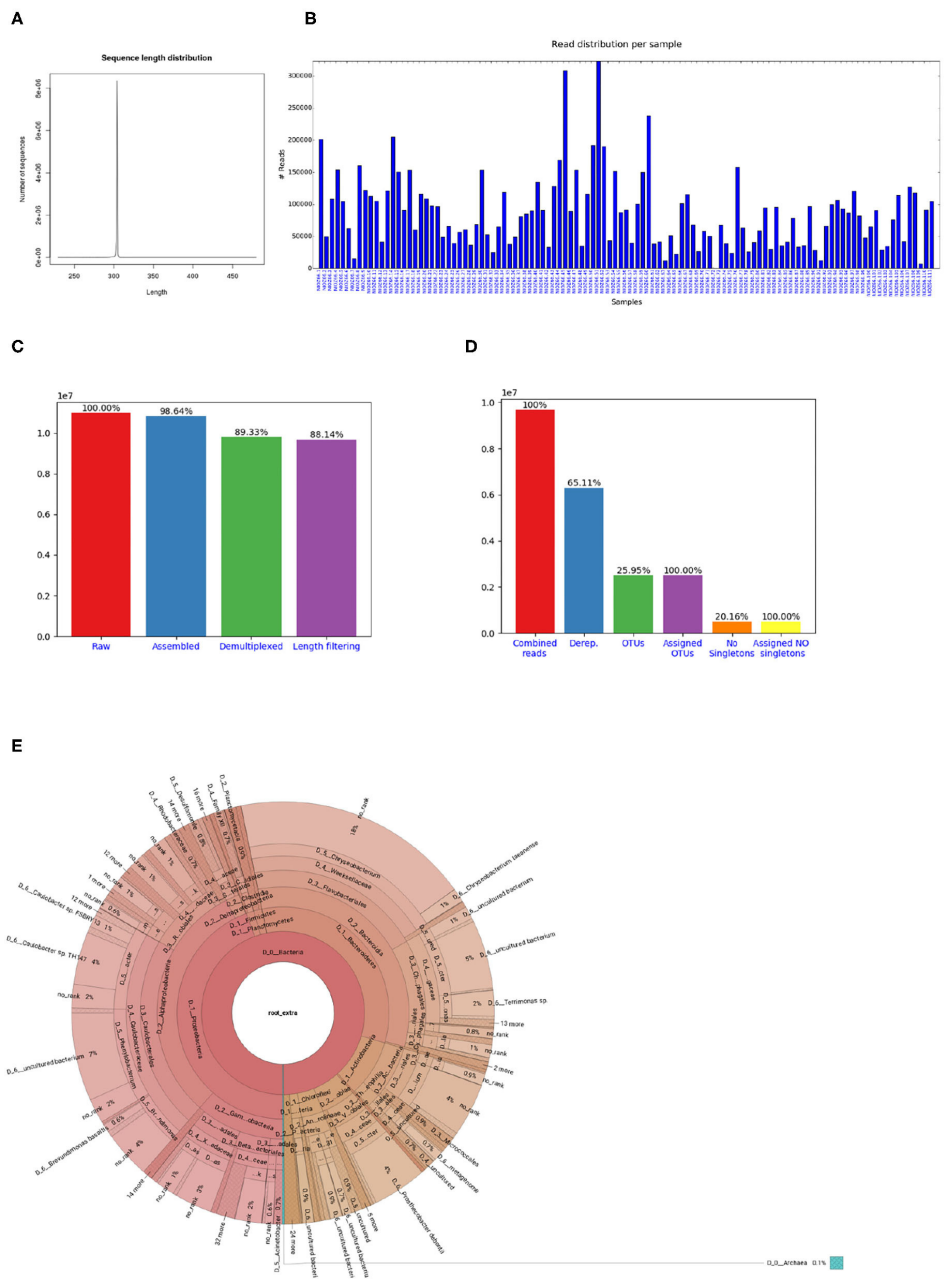
Figures shown in *Cascabel* reports. **(A)** Smoothed sequence length distribution after merging reads, for one library. The plot is meant to help making a sensible choice for sequence length filtering. **(B)** Number of sequences per sample. This histogram is part of the OTU report (including all libraries). **(C)** Number of sequences after individual pre-processing steps. “Assembled” refers to the number of raw read pairs which could be merged based on their overlap. “Demultiplexed” refers to the number of raw reads which could be assembled and assigned to a sample, and “Length filtering” indicates the number of raw reads passing the previous and the sequence length criteria. This plot is part of the library report. **(D)** Number of sequences after individual steps after potentially combining several libraries (total number of reads) and generating OTUs. “Derep.” indicates the number of dereplicated reads and their percentage relative to the total combined reads. “OTUs” is the total number of OTUs and the percentage is relative to the number of combined reads. “Assigned OTUs” is the number and percentage of OTUs with a taxonomic assignment. “No singletons” refers to the number and percentage of OTUs excluding singleton OTUs, and “Assigned NO singletons” is the number and percentage of singleton-free OTUs with a taxonomic assignation. The plot is part of the OTU report. **(E)** Krona chart for one sample. The krona charts are interactive and can be viewed with a web browser. Colors indicate the taxonomic groups to which the OTU was assigned. Each ring of the pie chart represents a different taxonomic level. An example of a full library report is shown in Supplementary Datasheet 3, and an OTU report is provided in Supplementary Datasheet 4.

If the library contains sequences from several samples, they are demultiplexed based on the barcode sequences provided in the barcode mapping file. To do so, *Cascabel* makes use of QIIME (Caporaso et al., 2010b) and a custom R script to (optionally) allow sequence errors in the barcodes. Demultiplexed data can also be stored in individual fastq files for further use outside the pipeline, e.g., for submitting data to public repositories. Optionally, *Cascabel* will align sequence reads against a reference sequence database to remove off-target reads and facilitate removing sequence adapters or primers or both. Adapter and primer sequences can be trimmed off with Cutadapt (Martin, 2011). Then, *Cascabel* generates a histogram of sequence lengths. In interactive mode, *Cascabel* shows the frequency of occurrence of each of the read lengths on the terminal and allows to change the minimum and maximum sequence length provided in the config file. The library report contains a smoothed histogram of the sequence lengths to validate the choice of the minimum and maximum sequence length (Figure 3A). Optionally, *Cascabel* identifies and removes chimeras either *de novo* based on sequence abundance or searching against the gold database provided by QIIME with the usearch61 algorithm (Edgar, 2010). The user can also provide different databases, such as SILVA (Quast et al., 2013) or PR2 (Guillou et al., 2013) to search for chimeras. Assembled and potentially filtered sequence reads from all samples are then concatenated into one fasta file. *Cascabel* generates a histogram to visualize the number of reads per sample for each of the libraries to assess whether the sequences are evenly spread across the samples (Figure 3B). Furthermore, the reports for each of the libraries contain a plot of the number and percentages of raw, assembled, demultiplexed and length filtered sequences (Figure 3C).

When working with large datasets, a dereplication step which collapses identical sequences into one representative sequence can drastically reduce computation time. *Cascabel* provides a custom rule based on VSEARCH (Rognes et al., 2016). *Cascabel*'s dereplication rule splits the data in two chunks and dereplicates them individually first, which, depending on how many duplicate sequences there are in the dataset, up to doubles the number of reads which can be dereplicated with the available memory. Then, the two chunks of dereplicated reads are merged and again dereplicated. To generate an OTU table, the dereplications are traced back by *Cascabel*.

*Cascabel* provides a range of popular methods to generate OTUs with or without a reference sequence database [Swarm (Mahé et al., 2015), sortmerna (Kopylova et al., 2012), mothur (Schloss et al., 2009), trie (Caporaso et al., 2010b), UCLUST/UCLUST_REF/USEARCH/USEARCH_REF (Edgar, 2010), prefix/suffix (Caporaso et al., 2010b), CD-HIT (Li and Godzik, 2006), and SUMACLUST (Kopylova et al., 2016)], some of these are executed by QIIME.

Then, representative sequences are chosen for each OTU (with options: random, longest, most_abundant, first) (Caporaso et al., 2010b). OTU and representative sequence picking methods provided by *Cascabel* are listed in Table 1. From the abundances of the OTU sequences within each of the samples, *Cascabel* creates an OTU abundance table. The OTUs can further be grouped at higher taxonomic levels depending on the desired resolution. An overview of the folder structure and main output files generated by *Cascabel* is given in Supplementary Datasheet 6.

Alternatively, *Cascabel* can perform Amplicon Sequence Variant (ASV) analysis with DADA2 (Callahan et al., 2016) for paired-end sequence data. In this case, *Cascabel* takes the demultiplexed fastq files and passes them to various R scripts which run sequence filtering, ASV identification, chimera detection and taxonomic assignment with DADA2. The main output of the ASV analysis are an ASV count table and ASV representative sequences. An example config file for an ASV analysis can be found in Supplementary Datasheet 2, and the ASV report for this analysis is shown in Supplementary Datasheet 5. The main output files of the ASV analysis are shown in Supplementary Datasheet 7.

*Cascabel* can process sequence data from any marker gene. *Cascabel* comes with taxonomic mapping files for 16S rRNA and 18S rRNA gene sequences from SILVA v132 (Quast et al., 2013), but the user can always choose to make use of a different public or a custom reference sequence database. *Cascabel* provides three different approaches to assign taxonomy to the representative sequences: VSEARCH, which performs global alignment of the target sequences against the reference database; BLAST, making use of BLAST+ (Camacho et al., 2009); QIIME, with methods BLAST (Altschul et al., 1990), UCLUST or the RDP classifier. Alternatively, any other public or custom database can be used for taxonomic annotation. If taxonomy is assigned with VSEARCH or BLAST, the user can choose to assign the sequences to the lowest common ancestor (LCA) with the stampa approach (https://github.com/frederic-mahe/stampa).

Subsequently, the user can opt to remove singletons, align representative sequences, filter the alignment and make a phylogenetic tree. To align representative sequences, *Cascabel* offers pynast (Caporaso et al., 2010a), mafft (Katoh and Standley, 2013), infernal (Nawrocki and Eddy, 2013), clustalw (Larkin et al., 2007), and muscle (Edgar, 2004). A phylogenetic tree can be generated with muscle, clustalw, raxml (Stamatakis, 2006) and fasttree (Price et al., 2009) (Table 1).

The last rule of *Cascabel* (the “target” rule) generates HTML and optional pdf reports with documentation, figures and tables summarizing the results of individual rules, as well as all software versions and parameter settings used. If more than one library was analyzed, there will be a report for each library as well as a report summarizing all libraries (otu_report or asv_report). Among other graphics, the otu_report shows the percentages and the total number of reads after filtering (“combined reads”), dereplicated reads, OTUs, OTUs assigned to a taxonomic level, OTUs excluding singletons (“no singletons”), and assigned OTUs excluding singletons (Figure 3D). The asv_report shows similar information in a table. Supplementary Datasheet 3 shows an example library report, Supplementary Datasheet 4 an otu_report, and Supplementary Datasheet 5 an asv_report. In addition, *Cascabel* generates an interactive Krona chart (Ondov et al., 2011) for the run which displays community composition for individual samples or the complete data set. The Krona chart shows the taxonomic assignments in an interactive HTML document composed of a multi-layered pie-chart and the user can zoom in and browse these different levels. An example is shown in Figure 3E.

A unique feature of *Cascabel* is its native handling of multiple analyses on the same dataset. Snakemake will not re-run a rule if the output file of that rule already exists, unless ––forcerun is used or the input file has a more recent date than the existing output file. This avoids unintentional over-writing of existing results, but also renders it impossible to keep results of multiple analyses on the same data in the same project. To allow multiple analyses within the same project, we implemented *Cascabel* with a “Run” parameter. Whenever the user changes the “Run” parameter, a new analysis will be performed (except for quality control on the raw data) and the results saved in a different “Run” folder. Each run has its own reports and is therefore fully documented and reproducible. Confusion about parameter settings for a specific analysis cannot happen.

The “Run” parameter is also useful to analyse data from primers which generate multiple fragments with different lengths. The data can then be analyzed with individual runs for each expected fragment length. For example, when using primers which amplify both bacterial/archaeal 16S and eukaryotic 18S sequences, albeit with different length, one run can target the shorter fragment and a second run the longer fragment. If the longer fragment generates non-overlapping read pairs, these can be analyzed with the “unpaired” workflow as indicated in the config file.

To facilitate comparing different taxonomy assignment approaches, the user can perform taxonomic assignments for the same run using different methods and the results will be saved in individual “taxonomy” folders. When starting a new taxonomic assignment, the existing OTU representative sequences are used so no processing time is wasted by performing the same upstream rules several times.

The user can make use of all intermediate files generated by individual rules, and most importantly the OTU or ASV table and representative sequences for follow-up analyses. To save disk space, the user can also opt to have *Cascabel* remove temporary files at the end of the analyses. For many rules, the user can pass additional parameters to the command or tool at hand using the “extra_params” parameter in the config file.

## 3. Results

### 3.1. *Cascabel* Example Analysis of a 16S Dataset

To demonstrate the functionality of *Cascabel*, we applied it to 16S rRNA gene amplicon data generated from water column samples taken from Lake Chala. *Cascabel* offers two routes of analysis: OTU and ASV analysis. Some rules apply to both routes, others only to one of them. This is indicated in the header of the rule in the config file by either “BOTH_WF,” “OTU_WF,” or “ASV_WF.” If choosing “OTU_WF,” for example, the “ASV_WF” rules and their parameter settings are ignored. We chose ‘ANALYSIS_TYPE: “OTU”’ here. After validating the input files, *Cascabel* proceeded with analyzing sequence data quality with FastQC (Andrews, 2010). In interactive mode, *Cascabel* will stop if more than a specified number of quality check modules failed, in non-interactive mode it will proceed. Next, we assembled read pairs, which is mandatory for paired-end data (rule “pear”). If the amplicon is so long that the forward and reverse read do not overlap, *Cascabel* can be run using the rule “UNPAIRED DATA WORK FLOW” and setting “UNPAIRED_DATA_PIPELINE” to true. After forward and reverse read assembly or merging, the quality of the assembled reads was again assessed with FastQC. Then, the sequencing library was demultiplexed based on the barcode sequences provided in the barcode mapping file (rules “write_dmx_files” and “extract_barcodes”). This step is optional to allow processing already demultiplexed data. We demultiplexed the Lake Chala data based on a sample barcode of 12 nucleotides at the beginning of the forward and the reverse read, using the “barcode_paired_stitched” configuration which merges the barcode sequence of the forward read with the barcode sequence of the reverse read. Barcode sequences were provided in a barcode mapping file, such as exemplified in Figure 1B. Individual barcodes were designed such that they have a nucleotide difference of at least three, however, we allowed only two mismatches in the merged barcode of 24 nucleotides to assign reads to samples to avoid false positive assignments due to sequencing errors. The demultiplexing rule can also save demultiplexed data in individual fastq files for further use outside the pipeline, e.g., for submitting data to public repositories, by setting the “create_fastq_files” parameter to “T” (true). During demultiplexing, technical sequences, such as primers can also be removed, and we did so for the Lake Chala data (primers are indicated in the config file, Supplementary Datasheet 1).

After demultiplexing, sequence chimeras can be removed based on a reference database, e.g., the gold database, and/or *de novo* based on sequence abundance, but we set this rule (“search_chimera”) to false. The next step is to filter out sequences with unexpected length. To facilitate setting length thresholds, *Cascabel* generates a smoothed histogram of observed sequence lengths, which is shown in the library report (Figure 3A). In interactive mode, *Cascabel* also shows the frequency of each of the read lengths on the terminal, and allows to change the minimum and maximum sequence length provided in the config file on the command line. For the analyzed example data, we filtered out sequences whose length differed more than 10 nucleotides from the average sequence length. Next, we dereplicated sequences which were identical over the full sequence length (rule “dereplicate”). This step is optional, but recommended to decrease the runtime of OTU clustering and avoid memory issues with very large datasets.

For OTU clustering, we chose UCLUST with a similarity threshold of 0.97, resulting in roughly 2.5 million OTUs (rule “cluster_OTUs”). We selected the longest sequence of an OTU as representative sequence to be used for taxonomic assignment of the OTU (rule “pick_representatives”). Then we used VSEARCH to assign taxonomy to the representative sequences based on the SILVA database (SILVA version 132, rule “assign_taxonomy”). From the abundances of the OTU sequences within each of the samples, *Cascabel* creates an OTU abundance table in BIOM and plain text format (rule “make_otu_table”). The OTUs were also summarized at different taxonomic levels making use of the rule “summarize_taxa.” Subsequently, we removed singletons (rule “filter_otu”), aligned representative sequences (“align_rep_seqs”), filtered the alignment (“filter_alignment”), and made a phylogenetic tree (“make_tree”). Removing singletons reduced the number of OTUs in the analyzed dataset to roughly 500, 000. To align representative sequences, we used pynast and fasttree to generate a phylogenetic tree. Finally, *Cascabel* generated a Krona chart, and HTML and pdf reports of the analysis, documenting all software and parameters used. Supplementary Datasheet 1 contains the config file with parameter settings for the analysis described above.

The total runtime of *Cascabel* using the OTU workflow on the Lake Chala dataset was 14.5 h. Currently, we experience a bottleneck in the runtime of the barcode error correction, which took 10.25 h on this large dataset and will be improved in future versions of *Cascabel*. We also ran the ASV workflow on the same data (config file shown in Supplementary Datasheet 2), which took 13.2 h in total, with again barcode error correction being the most time-consuming step (10.25 h). Running *Cascabel* assigning reads with perfectly matching barcodes only would take 4.25 h for the OTU workflow and 2.95 h for the ASV workflow.

### 3.2. Analyses of Mock Datasets

We evaluated the results of the individual runs in terms of the number of genera identified correctly (true positives), the number of genera missed (false negatives) and the number of genera identified which were not part of the mock community (false positives). We evaluated all runs with respect to true and false positives, and show the individual true and false positive genera for a selection of the runs which we performed on the mock datasets. The selection included at least one run using UCLUST, one using Swarm and one ASV run with DADA2. We also varied Swarm parameters, reference databases, clustering thresholds, and chimera detection, but evaluating all possible combinations of parameters would not be feasible. An overview of the runs performed and the evaluation in terms of true and false positives, false negatives, precision, recall and F1 statistic (harmonic mean of precision and recall), is shown in Supplementary Datasheet 8. While on the 16S mock dataset, all of UCLUST, swarm and DADA2 had a very good recall rate of 0.95, the OTU/ASV clustering methods had different numbers of false positive predictions. DADA2 had the lowest number of false positives (1), followed by Swarm (14–27) and UCLUST (21–25). Therefore, DADA2 performed best in terms of precision (0.95) and F1 statistic (0.95). The 18S mock dataset was more challenging than the 16S dataset for all combinations of methods tested. UCLUST with a similarity threshold of 0.97 and VSEARCH for taxonomy assignment using SILVA v138 performed best in terms of recall (0.92). However, DADA2 performed best concerning precision (1.0) and the harmonic mean of precision and recall (0.8). On the ITS dataset, the best performance was shown by Swarm using *d* = 2 and VSEARCH for taxonomy assignation, with an F1 statistic of 0.92. This run also showed the best recall (0.89). The highest precision was achieved by UCLUST (1.0), however, with a very low F1 statistic (0.19). The performance of DADA2 was lower than the one of Swarm, with an F1 statistic of 0.8. Thus, we observed substantial differences between different methods and parameter settings, and there was no one setting that would perform best on all three datasets. On the contrary, the best results were obtained with different methods and parameter settings for different marker genes. These results confirm that it is important to have a flexible pipeline to adapt it to the needs of the dataset at hand, but also that it is important to include a mock community ideally in every sequencing run that is performed to allow making informed choices about method and parameter selection. We also compared the different clustering algorithms in terms of runtime. The 16S mock community consisted of 207,197 paired-end reads of 300 nucleotides each, considerably smaller than the Lake Chala dataset, and therefore the analyses were much faster. The analysis with UCLUST (config_4.yaml) took 43 min and 18 s, of which 38 min and 42 s were spent on searching chimeras *de novo*. Swarm needed 5 min and 16 s for a run including chimera search against a reference database (config_12.yaml), searching chimeras took 1 min and 24 s of the total time. A DADA2 run (config_14.yaml) needed 8 min and 52 s including DADA2's own chimera search method.

## 4. Discussion

*Cascabel* has been developed at the Royal Netherlands Institute for Sea Research (NIOZ) to facilitate, unify and easily track data provenance of amplicon sequence data analyses. Apparent advantages of using this pipeline compared to custom scripts are that the individual steps of the pipeline have been tested by many members of the community at the NIOZ who are experienced in amplicon sequencing data analyses (van Bleijswijk et al., 2015; Balzano et al., 2018; Besseling et al., 2019; Klunder et al., 2019), and therefore should contain fewer mistakes than scripts that were written for a specific analysis by one person. Moreover, community knowledge and experience have created a workflow which is probably more comprehensive and powerful than one that was created by a single person. In addition, the availability of the pipeline has facilitated comparing and integrating research results from different data sets generated at the NIOZ because scientists can agree on certain settings and reference database versions and the pipeline guarantees that the analyses are performed in the same way. Because *Cascabel* keeps track of data provenance, documenting the process of analyzing the data to generate results, it also facilitates preparing research manuscripts. While most of the scientific journals request the raw sequencing data to be submitted to a public repository for many years already, also reporting data provenance becomes more important. The journal “Nature,” for example, requires authors to make materials, data, code, and associated protocols available (www.nature.com, 2019). *Cascabel* facilitates providing data, code and protocols. Public sequence repositories often require the raw data to be submitted per sample, but sample demultiplexing typically takes place after merging read pairs such that the raw data cannot be recovered. Therefore, *Cascabel* demultiplexes the raw data in parallel to the analyses such that it is ready for public data repository deposition. The code of Cascabel is open source and all analyses are protocolled in the reports and config file, complying with the rules for reproducible computational research described by Sandve et al. (2013).

DNA sequencing technology, algorithms and analysis approaches are constantly evolving. It is logical that pipelines lag behind with the most recent developments because it takes time to test and integrate new modules. Because *Cascabel* is a Snakemake workflow, it is flexible and easy to extend to encompass more or alternative rules. We are constantly working on extending the range of applications and making new methods available. For the sake of consistency, we deliberately keep older methods to allow users to compare runs using their familiar algorithm with newer algorithms and to compare or integrate new data with data generated previously.

The task of generating biological meaningful microbial community profiles from amplicon sequence data is far from trivial, and we believe that there is not one best strategy for data analysis. Based on the environment investigated and the scientific question, desired taxonomic resolution may differ. Therefore, we do not want to promote any optimal settings of the tools used by *Cascabel*. We do, however, provide some guidance by making pre-configured config files available, but advise any user to check them carefully and modify them to their needs. Our analyses on public mock datasets have shown that the optimal method may depend on the marker gene and the dataset at hand. Therefore, we advise users to evaluate their favorite configurations for an analysis on mock datasets and ideally include a mock community in their own sequencing projects.

*Cascabel* provides reference databases for taxonomy assignment and chimera detection, but the user can always supply a different database and specify that in the config file. Moreover, *Cascabel* is not limited to Illumina sequence data that we used for demonstration purposes, but can handle sequence data from other technologies which produce short reads from amplicons as well (e.g., Ion Torrent). With some minor modifications, *Cascabel* can even be used to analyze long read amplicon sequence data.

Galaxy (Afgan et al., 2018) is a user-friendly web-based alternative to *Cascabel* which offers interfaces to VSEARCH and mothur executables. Having a medium-sized user group at the institute, we did not want to overload a public server and setting up and maintaining our own server would also need resources that we preferred to allocate to the development of a workflow for which we have full control and flexibility. With *Cascabel* being invoked from the command line, the user can make use of the full potential that Snakemake has to offer, e.g., ––prioritize to force the execution of specific rules prior to others when distributing tasks across computing resources, ––until to run the pipeline up to a specific rule, ––summary, which shows the rules executed so far and ––dag which shows the rules executed and the ones yet to be done in a directed acyclic graph. Moreover, we consider *Cascabel*'s report an essential element to move forward in terms of user-friendly data provenance and reproducibility.

We have presented *Cascabel*, an open source pipeline to analyze amplicon sequence data based on the workflow engine Snakemake. The pipeline can be easily installed using Anaconda or Miniconda, comes with documentation, a wiki, and a test dataset on github and can be executed by users with basic command line skills. At the same time, *Cascabel* is flexible, offering alternative options for most of the steps and supporting custom reference databases, and can easily be modified and extended by users with computational skills. Moreover, all analyses performed with *Cascabel* are fully documented and reproducible. We believe that *Cascabel* will prove to be useful to scientist who need more flexibility and throughput than provided by tools based on web servers, but do not want to or cannot generate their own command-line based workflow.

## 5. Methods

### 5.1. Sampling, DNA Extraction, and Sequencing of Example Dataset

Suspended particulate matter (SPM) was collected from the water column of Lake Chala, a lake situated on the border of Kenya and Tanzania, east of Mount Kilimanjaro in Africa, from September 2013 to May 2014 from a total of 111 samples as described in van Bree et al. (2018). DNA was extracted from 1/32 section of the filters on which SPM was collected by using the PowerSoil DNA extraction kit (Mo Bio Laboratories, Carlsbad, CA, USA).

The V4 region of the 16S rRNA gene was amplified with the primers forward:

515F: GTGYCAGCMGCCGCGGTAA (Parada et al., 2016) and reverse:

806RB: GGACTACNVGGGTWTCTAAT (Apprill et al., 2015). We made use of 12 nucleotide Golay barcodes at the beginning of the forward and the reverse read. Paired-end sequencing of 250 nt was performed on an Illumina MiSeq instrument (Illumina, San Diego, CA) using the Truseq DNA nano LT kit for library preparation and V3 sequencing chemistry at the sequencing facility of the University of Utrecht (USEQ), the Netherlands. The dataset contains a total of 10, 979, 168 paired-end sequence reads. The data is publicly available at NCBI, BioProject PRJNA526242. Sample and run identifiers of the samples used are listed in Supplementary Datasheet 9.

### 5.2. Analysis of Example Dataset

Starting from the config file template for paired-end sequencing data (config.otu.double_bc.yaml, provided on github: https://github.com/AlejandroAb/CASCABEL), we supplied the file paths to the raw sequence data (fastq files) in the “GENERAL PARAMETERS SECTION” (subsection “INPUT FILES”). Note that fastq files can also be provided as gzipped files, then in the “INPUT TYPE” section of the general parameter section, the parameter *gzip_input* needs to be set to “T” (True). The barcode mapping file was passed to *Cascabel via* the “metadata” parameter in the subsection “INPUT FILES” of the config file. In the “GENERAL PARAMETERS SECTION,” we chose a project name (“CascabelTest”) and set the “RUN” parameter to “report_test,” *Cascabel* then used these names to generate a project folder and a run folder. All settings and parameters chosen to analyze the example data set are documented in the config files (Supplementary Datasheets 1, 2) and the reports (Supplementary Datasheets 3–5). The reports also contain software versions of third-party tools incorporated in *Cascabel*.

### 5.3. Analyses of Mock Community Datasets

To show the flexibility and assess the performance of running *Cascabel* with different methods and parameter settings, we analyzed three published mock community datasets with multiple *Cascabel* runs. We chose one dataset consisting of 16S rRNA data, one of 18S rRNA data and one of ITS sequences. The 16S rRNA dataset is part of the public resource project for bioinformatics benchmark data, Mockrobiota (Bokulich et al., 2016). The mock community is composed of 20 evenly distributed bacterial strains as described in Gohl et al. (2016). For the ITS marker gene, we used a dataset of Bakker (2018), composed of 19 fungal species with staggered abundances, intended to mimic the abundance distribution of natural microbial communities. Finally, for the 18S rRNA marker gene, we selected a mock community composed of 12 algal species across five major divisions of eukaryotic microalgae (Bradley et al., 2016). More information about the selected datasets, sample accessions, links to the rawdata and different parameters used to run *Cascabel* can be found in Supplementary Datasheet 8. The config files of the individual runs are available in Supplementary Datasheet 10 and at *Cascabel's* test data repository (https://github.com/AlejandroAb/CASCABEL-Test/tree/master/mock_analysis). Fastq files were downloaded from the European Nucleotide Archive (ENA) using an in-house download tool (https://github.com/AlejandroAb/ENA-downloader-tool).

## Data Availability Statement

The datasets generated for this study can be found in the SRA of NCBI, BioProject PRJNA526242.

## Author Contributions

AA implemented the pipeline, with contributions from JE. MB, LV, SB, and HW tested the pipeline. AA, JB, and JE designed the pipeline, with contributions from LV. JE wrote the manuscript. All authors contributed to and approved the final version of the manuscript.

## Conflict of Interest

The authors declare that the research was conducted in the absence of any commercial or financial relationships that could be construed as a potential conflict of interest.

## Supplementary Material

Supplementary Datasheet 1—Config file for OTU analysis The config file which was used to run an OTU analysis with *Cascabel* on the example 16S dataset.

Supplementary Datasheet 2—Config file for ASV analysis The config file which was used to run an ASV analysis with *Cascabel* on the example 16S dataset.

Supplementary Datasheet 3—*Cascabel* library report PDF report generated by *Cascabel* for the example 16S sequencing data analysis. The report contains the names and locations of all input and output files, names and short description of the modules (“rules”) and parameters which were used in the analysis. In addition, graphics summarize the data in terms of sequence output and number of sequences left after each step of the analysis.

Supplementary Datasheet 4—*Cascabel* OTU report PDF report describing the OTU analysis of the example 16S sequencing data. It contains the names and locations of all input and output files, names and short description of the modules (“rules”) and parameters which were used in the analysis. In addition, graphics summarize the data in terms of sequence output per sample, number of OTUs and taxonomic composition.

Supplementary Datasheet 5—*Cascabel* ASV report PDF report describing the ASV analysis of the example 16S sequencing data. It contains the names and locations of all input and output files, names and short description of the modules (“rules”) and parameters which were used in the analysis. In addition, the data is summarized in terms of sequence output per sample, number of ASVs and taxonomic composition.

Supplementary Datasheet 6—Folder structure of results of an OTU analysis This folder structure is generated for the output of an OTU analysis performed with *Cascabel*. Note that only the main files are displayed, log files and temporary files are not shown.

Supplementary Datasheet 7—Folder structure of results of an ASV analysis This folder structure is generated for the output of an ASV analysis performed with *Cascabel*. Note that only the main files are displayed, log files and temporary files are not shown.

Supplementary Datasheet 8—Mock community datasets and evaluation List of mock community datasets used, including NCBI BioProject and SRA accession numbers. Overview of *Cascabel* runs performed on the mock community data and evaluation of the performance of the individual runs.

Supplementary Datasheet 9—SRA identifiers of PRJNA526242 Table spreadsheet indicating the sample, experiment and run identifiers of BioProject PRJNA526242 to download the raw data used for the worked example from the NCBI Sequence Read Archive (SRA).

Supplementary Datasheet 10—Config files of mock community analyses Config files of the *Cascabel* runs performed on the mock community datasets as listed in Supplementary Datasheet 8.
