# Supplementary material for: Cascabel: A Scalable and Versatile Amplicon Sequence Data Analysis Pipeline Delivering Reproducible and Documented Results
Source: Front Genet. 2020 Nov 20;11:489357. doi: 10.3389/fgene.2020.489357 (PMC7718033; doi:10.3389/fgene.2020.489357)
Supplement: Supplementary Datasheet 3—Cascabel library report — PDF report generated by Cascabel for the example 16S sequencing data analysis. The report contains the names and locations of all input and output files, names and short description of the modules (“rules”) and parameters which were used in the analysis. In addition, graphics summarize the data in terms of sequence output and number of sequences left after each step of the analysis. [file Data_Sheet_3.PDF]

snakemake report

Amplicon Analysis Report for Library: LakeChala

CASCABEL is designed to run amplicon sequence analysis across single or multiple read libraries.

The objective of this pipeline is to create different output files which allow the user to explore data in a simple and meaningful way, as well as facilitate downstream analysis, based on the generated output files.

Another aim of **CASCABEL** is also to encourage the documentation process, by creating this report in order to assure data analysis reproducibility.

User description: Example for single library run with Cascabel using the OTU analysis workflow

Following you can see all the steps that were taken in order to get the final results of the pipeline.

Raw Data

The raw data for this library can be found at:

- FW raw reads: CascabelTest/samples/LakeChala/rawdata/fw.fastq

- RV raw reads: CascabelTest/samples/LakeChala/rawdata/rv.fastq

Number of total reads: 10979168

Quality Control

Evaluate quality on raw reads.

Tool: [FastQC]

Version: **FastQC v0.11.7**

Command:

```
fastqc CascabelTest/samples/LakeChala/rawdata/fw.fastq CascabelTest/samples/LakeChala/rawdata/rv.fastq --extract -o
CascabelTest/samples/LakeChala/qc/
```

You can follow the links below, in order to see the complete FastQC report:

- FastQC for sample LakeChala_1: FQ1

- FastQC for sample LakeChala_2: FQ2

Benchmark info:

s	max_rss	max_vms	max_uss	max_pss	io_in	io_out	mean_load
260.42	347.41	3914.08	346.02	346.27	11737.91	2.51	0.00

Read pairing

Align paired end reads and merge them into one single sequence in case they overlap.

Tool: [PEAR]

version: **PEAR v0.9.10 [May 30, 2016] - [+bzlib +zlib]**

Command:

```
pear -f CascabelTest/samples/LakeChala/rawdata/fw.fastq -r CascabelTest/samples/LakeChala/rawdata/rv.fastq -t 100 -v 10 -j 6 -p
0.05 -o CascabelTest/runs/report_test_otu/LakeChala_data/peared/seqs >
CascabelTest/runs/report_test_otu/LakeChala_data/peared/seqs.assembled.fastq
```

Output files:

- Merged reads: CascabelTest/runs/report_test_otu/LakeChala_data/peared/seqs.assembled.fastq

- Log file: CascabelTest/runs/report_test_otu/LakeChala_data/peared/pear.log

Number of peared reads: 10829329 = 98.64%

Benchmark info:

s	max_rss	max_vms	max_uss	max_pss	io_in	io_out	mean_load
1522.39	190.44	748.05	188.66	188.70	0.00	7178.65	0.00

Peared FastQC Analysis

Check the quality of the reads after assembly.

Tool: [FastQC]

Version: **FastQC v0.11.7**

Command:

```
fastqc CascabelTest/runs/report_test_otu/LakeChala_data/peared/seqs.assembled.fastq --extract -o
CascabelTest/runs/report_test_otu/LakeChala_data/peared/qc
```

Output files:

- FastQC report: CascabelTest/runs/report_test_otu/LakeChala_data/peared/qc/seqs.assembled_fastqc.html FQ_Report

Benchmark info:

s	max_rss	max_vms	max_uss	max_pss	io_in	io_out	mean_load
158.33	279.55	3831.53	277.77	277.92	6210.00	0.14	0.00

Extract barcodes

Extract the barcodes used to identify individual samples.

Tool: [QIIME] - extract_barcodes.py

Version: **extract_barcodes.py 1.9.1**

Command:

```
extract_barcodes.py -f CascabelTest/runs/report_test_otu/LakeChala_data/peared/seqs.assembled.fastq -c
barcode_paired_stitched --bc1_len 12 --bc2_len 12 -o CascabelTest/runs/report_test_otu/LakeChala_data/barcodes/
```

Output files:

- Fastq file with barcodes: CascabelTest/runs/report_test_otu/LakeChala_data/barcodes/barcodes.fastq

- Fastq file with the reads: CascabelTest/runs/report_test_otu/LakeChala_data/barcodes/reads.fastq

Benchmark info:

s	max_rss	max_vms	max_uss	max_pss	io_in	io_out	mean_load
292.82	119.07	5616.20	116.45	116.67	7049.73	7631.81	0.00

Correct Barcodes

Try to correct the barcode from unassigned reads.

Maximum number of mismatches **2**.

Tool: CASCABEL's R script

Command:

```
Rscript Scripts/errorCorrectBarcodes.R $PWD CascabelTest/metadata/sampleList_mergedBarcodes_LakeChala.txt
CascabelTest/runs/report_test_otu/LakeChala_data/barcodes/barcodes.fastq 2
```

Output file:

- Barcode corrected file: CascabelTest/runs/report_test_otu/LakeChala_data/barcodes/barcodes.fastq_corrected

Benchmark info:

s	max_rss	max_vms	max_uss	max_pss	io_in	io_out	mean_load
18380.04	911.42	1465.00	901.28	903.67	1117.33	1122.13	0.00

Demultiplexing

For library splitting, also known as demultiplexing, Cascabel performs several steps to assign fragments in the original as well as reverse orientation to the correct sample.

Split samples from Fastq file

Tool: [QIIME] - split_libraries_fastq.py

version: **split_libraries_fastq.py 1.9.1**

Command:

```
split_libraries_fastq.py -m CascabelTest/metadata/sampleList_mergedBarcodes_LakeChala.txt -i
CascabelTest/runs/report_test_otu/LakeChala_data/barcodes/reads.fastq -o
CascabelTest/runs/report_test_otu/LakeChala_data/splitLibs -b
CascabelTest/runs/report_test_otu/LakeChala_data/barcodes/barcodes.fastq_corrected -q 19 -r 5 --retain_unassigned_reads --
barcode_type 24
```

Benchmark info:

s	max_rss	max_vms	max_uss	max_pss	io_in	io_out	mean_load
567.50	460.38	5570.93	457.73	457.96	7841.59	4481.81	0.00

Retain assigned reads

Command:

```
cat CascabelTest/runs/report_test_otu/LakeChala_data/splitLibs/seqs.fna | grep -P -A1 "(?!>Unass)^>" | sed '/^--$/d' >
CascabelTest/runs/report_test_otu/LakeChala_data/splitLibs/seqs.assigned.fna
```

Create file with only unassigned reads

Command:

```
cat CascabelTest/runs/report_test_otu/LakeChala_data/splitLibs/seqs.fna | grep "^>Unassigned" | sed 's/>Unassigned_[0-9]* /@/g'
| sed 's/ .*//' | grep -F -w -A3 -f - CascabelTest/runs/report_test_otu/LakeChala_data/peared/seqs.assembled.fastq | sed '/^--$/d'
>CascabelTest/runs/report_test_otu/LakeChala_data/splitLibs/unassigned.fastq
```

Reverse complement unassigned reads

Tool: [Vsearch]

version: **vsearch v2.8.0_linux_x86_64, 754.8GB RAM, 144 cores**

Command:

```
vsearch --fastx_revcomp CascabelTest/runs/report_test_otu/LakeChala_data/splitLibs/unassigned.fastq --fastqout
CascabelTest/runs/report_test_otu/LakeChala_data/splitLibs/unassigned.reversed.fastq
```

Barcode extraction for reverse complemented, unassigned reads

Tool: [QIIME] - extract_barcodes.py

Version: **extract_barcodes.py 1.9.1**

Command:

```
extract_barcodes.py -f CascabelTest/runs/report_test_otu/LakeChala_data/splitLibs/unassigned.reversed.fastq -c
barcode_paired_stitched --bc1_len 12 --bc2_len 12 -o CascabelTest/runs/report_test_otu/LakeChala_data/barcodes_unassigned/
```

Correct reverse complemented barcodes

Maximum number of mismatches **2**.

Tool: CASCABEL's R script

Command:

```
Rscript Scripts/errorCorrectBarcodes.R $PWD CascabelTest/metadata/sampleList_mergedBarcodes_LakeChala.txt
CascabelTest/runs/report_test_otu/LakeChala_data/barcodes_unassigned/barcodes.fastq_corrected 2
```

Output file:

- Barcode corrected file: CascabelTest/runs/report_test_otu/LakeChala_data/barcodes/barcodes.fastq_corrected

Benchmark info:

s	max_rss	max_vms	max_uss	max_pss	io_in	io_out	mean_load
17103.24	870.19	1423.86	860.14	862.60	755.34	759.40	0.00

Split reverse complemented reads

Tool: [QIIME] - extract_barcodes.py

Version: **extract_barcodes.py 1.9.1**

Command:

```
split_libraries_fastq.py -m CascabelTest/metadata/sampleList_mergedBarcodes_LakeChala.txt -i
CascabelTest/runs/report_test_otu/LakeChala_data/barcodes_unassigned/reads.fastq -o
CascabelTest/runs/report_test_otu/LakeChala_data/splitLibsRC -b
CascabelTest/runs/report_test_otu/LakeChala_data/barcodes_unassigned/barcodes.fastq_corrected -q 19 -r 5 --barcode_type 24
```

Benchmark info:

s	max_rss	max_vms	max_uss	max_pss	io_in	io_out	mean_load
567.50	460.38	5570.93	457.73	457.96	7841.59	4481.81	0.00

Output files:

- Text histogram with the length of the fw reads: CascabelTest/runs/report_test_otu/LakeChala_data/splitLibs/histograms.txt

- Log file for the fw reads: CascabelTest/runs/report_test_otu/LakeChala_data/splitLibs/split_library_log.txt

- Text histogram with the length of the rv reads: CascabelTest/runs/report_test_otu/LakeChala_data/splitLibsRC/histograms.txt

- Log file for the rv reads: CascabelTest/runs/report_test_otu/LakeChala_data/splitLibsRC/split_library_log.txt

- Fasta file with unassigned reads: CascabelTest/runs/report_test_otu/LakeChala_data/splitLibsRC/seqs.unassigned.fna

Number of reads assigned on FW: 3235588 = 29.88% of the peared reads

Number of reads assigned on RVC: 3267197 = 30.17% of the peared reads

Generate single sample fastq files

Create single fastq files per samples (based on the raw data without applying any filtering).

Tool: CASCABEL's Java program

Command:

```
java -cp Scripts DemultiplexQiime --fasta -d CascabelTest/runs/report_test_otu/LakeChala_data/seqs_fw_rev_accepted.fna -o
CascabelTest/runs/report_test_otu/LakeChala_data/demultiplexed/ -r1 CascabelTest/samples/LakeChala/rawdata/fw.fastq -r2
CascabelTest/samples/LakeChala/rawdata/fw.fastq
```

The demultiplexed files are located at:

- demultiplexed directory: CascabelTest/runs/report_test_otu/LakeChala_data/demultiplexed/

- summary file: CascabelTest/runs/report_test_otu/LakeChala_data/demultiplexed/summary.txt

Benchmark info:

s	max_rss	max_vms	max_uss	max_pss	io_in	io_out	mean_load
1769.02	4198.51	82975.27	4210.87	4210.96	26.74	717.73	0.00

Combine reads

Concatenate forward and reverse reads.

Command:

```
cat CascabelTest/runs/report_test_otu/LakeChala_data/splitLibs/seqs.assigned.fna
CascabelTest/runs/report_test_otu/LakeChala_data/splitLibsRC/seqs.assigned.fna >
CascabelTest/runs/report_test_otu/LakeChala_data/seqs_fw_rev_accepted.fna
```

Output files:

- Fasta file with combined reads: CascabelTest/runs/report_test_otu/LakeChala_data/seqs_fw_rev_accepted.fna

- Total number of acepted reads: 6502785 = 60.05% of the peared reads or 59.23% of the raw reads.

Benchmark info:

s	max_rss	max_vms	max_uss	max_pss	io_in	io_out	mean_load
5.13	2.96	217.00	1.34	1.35	0.00	2740.52	0.00

Remove too long and too short reads

Remove very short and long reads, with lengths more than some standard deviation below or above the mean to be short or long respectively

- Minimun length expected (shorts): 282

- Maximun length expected (longs): 302

Command:

```
awk '!/^>/ { next } { getline seq } length(seq) > shorts && length(seq) < longs { print $0 "n" seq }'
CascabelTest/runs/report_test_otu/LakeChala_data/seqs_fw_rev_accepted.fna >
CascabelTest/runs/report_test_otu/LakeChala_data/seqs_fw_rev_filtered.fasta
```

Sequence distribution before remove reads

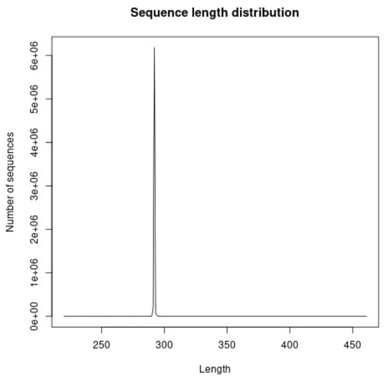

Output file:

- Fasta file with correct sequence length: CascabelTest/runs/report_test_otu/LakeChala_data/seqs_fw_rev_filtered.fasta

Total number of reads after length filtering: 6495704

Percentage of reads vs raw reads: 59.16%

Percentage of reads vs demultiplexed reads: 99.89%

Benchmark info:

s	max_rss	max_vms	max_uss	max_pss	io_in	io_out	mean_load
15.45	22.05	471.52	14.38	15.65	0.02	2613.55	0.00

Sample distribution

Sample	Seqs	prc.	Sample	Seqs	prc.	Sample	Seqs	prc.	Sample	Seqs	prc.
NIOZ66.1	129627	2.00	NIOZ66.29	43053	0.66	NIOZ66.57	13373	0.21	NIOZ66.85	26248	0.40
NIOZ66.2	31669	0.49	NIOZ66.30	112853	1.74	NIOZ66.58	67364	1.04	NIOZ66.86	55497	0.85
NIOZ66.3	63835	0.98	NIOZ66.31	38895	0.60	NIOZ66.59	100895	1.55	NIOZ66.87	19299	0.30
NIOZ66.4	117095	1.80	NIOZ66.32	4965	0.08	NIOZ66.60	41181	0.63	NIOZ66.88	15351	0.24
NIOZ66.5	128837	1.98	NIOZ66.33	38843	0.60	NIOZ66.61	27370	0.42	NIOZ66.89	71372	1.10
NIOZ66.6	40967	0.63	NIOZ66.34	84597	1.30	NIOZ66.62	29453	0.45	NIOZ66.90	18212	0.28
NIOZ66.7	4929	0.08	NIOZ66.35	17368	0.27	NIOZ66.63	6101	0.09	NIOZ66.91	3695	0.06
NIOZ66.8	122472	1.89	NIOZ66.36	29387	0.45	NIOZ66.64	32710	0.50	NIOZ66.92	42290	0.65
NIOZ66.9	87521	1.35	NIOZ66.37	50014	0.77	NIOZ66.65	14893	0.23	NIOZ66.93	79459	1.22
NIOZ66.10	80962	1.25	NIOZ66.38	50920	0.78	NIOZ66.66	76242	1.17	NIOZ66.94	87181	1.34
NIOZ66.11	71434	1.10	NIOZ66.39	65405	1.01	NIOZ66.67	81637	1.26	NIOZ66.95	68980	1.06
NIOZ66.12	8829	0.14	NIOZ66.40	103828	1.60	NIOZ66.68	2178	0.03	NIOZ66.96	66115	1.02
NIOZ66.13	82058	1.26	NIOZ66.41	66426	1.02	NIOZ66.69	15958	0.25	NIOZ66.97	86801	1.34
NIOZ66.14	140946	2.17	NIOZ66.42	20937	0.32	NIOZ66.70	40119	0.62	NIOZ66.98	55920	0.86
NIOZ66.15	105775	1.63	NIOZ66.43	75438	1.16	NIOZ66.71	31500	0.48	NIOZ66.99	31005	0.48
NIOZ66.16	60172	0.93	NIOZ66.44	138755	2.14	NIOZ66.72		ND	NIOZ66.100	36810	0.57
NIOZ66.17	119359	1.84	NIOZ66.45	180218	2.77	NIOZ66.73	45179	0.70	NIOZ66.101	68419	1.05
NIOZ66.18	42666	0.66	NIOZ66.46	54947	0.85	NIOZ66.74	29017	0.45	NIOZ66.102	19543	0.30
NIOZ66.19	84569	1.30	NIOZ66.47	118656	1.83	NIOZ66.75	17409	0.27	NIOZ66.103	12549	0.19
NIOZ66.20	81017	1.25	NIOZ66.48	16694	0.26	NIOZ66.76	94318	1.45	NIOZ66.104	51442	0.79
NIOZ66.21	68616	1.06	NIOZ66.49	82504	1.27	NIOZ66.77	43010	0.66	NIOZ66.105	86163	1.33
NIOZ66.22	70670	1.09	NIOZ66.50	157388	2.42	NIOZ66.78	14150	0.22	NIOZ66.106	14479	0.22
NIOZ66.23	27319	0.42	NIOZ66.51	247913	3.82	NIOZ66.79	20855	0.32	NIOZ66.107	91360	1.41
NIOZ66.24	39338	0.61	NIOZ66.52	146907	2.26	NIOZ66.80	35417	0.55	NIOZ66.108	79706	1.23
NIOZ66.25	22692	0.35	NIOZ66.53	45991	0.71	NIOZ66.81	69235	1.07	NIOZ66.109	4999	0.08
NIOZ66.26	22802	0.35	NIOZ66.54	114135	1.76	NIOZ66.82	9607	0.15	NIOZ66.110	66703	1.03
NIOZ66.27	33431	0.51	NIOZ66.55	60575	0.93	NIOZ66.83	70693	1.09	NIOZ66.111	67129	1.03
NIOZ66.28	25271	0.39	NIOZ66.56	46934	0.72	NIOZ66.84	9689	0.15			

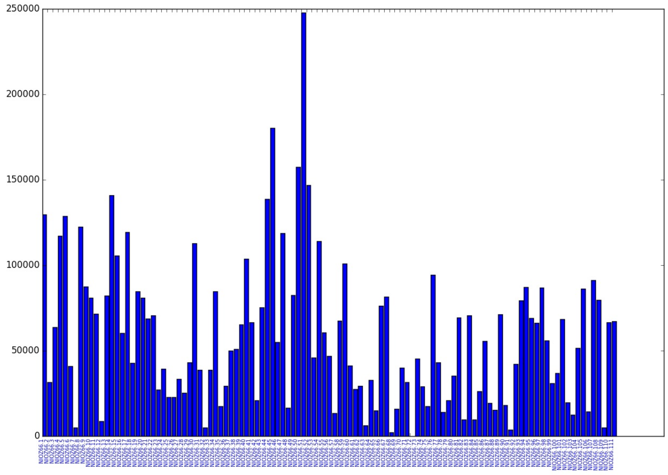

The previous chart shows the number of clean reads per sample. The bars are sorted from left to right, according to the metadata input file.

To see more details about the number of reads per sample in this library, please refer to the file: CascabelTest/runs/report_test_otu/LakeChala_data/seqs_fw_rev_filtered.dist.txt

Final counts

Following you can see the final read counts:

File description	Location	Number of reads	Prc(%) vs raw
Raw reads	CascabelTest/samples/LakeChala/rawdata/*.fq	10979168.0	100.00%
Assembled reads	CascabelTest/runs/report_test_otu/LakeChala_data/peared/seqs.assembled.fastq	10829329.0	98.64%
Demultiplexed reads	CascabelTest/runs/report_test_otu/LakeChala_data/seqs_fw_rev_accepted.fna	6502785	59.23%
Length filtered	CascabelTest/runs/report_test_otu/LakeChala_data/seqs_fw_rev_filtered.fasta	6495704	59.16%

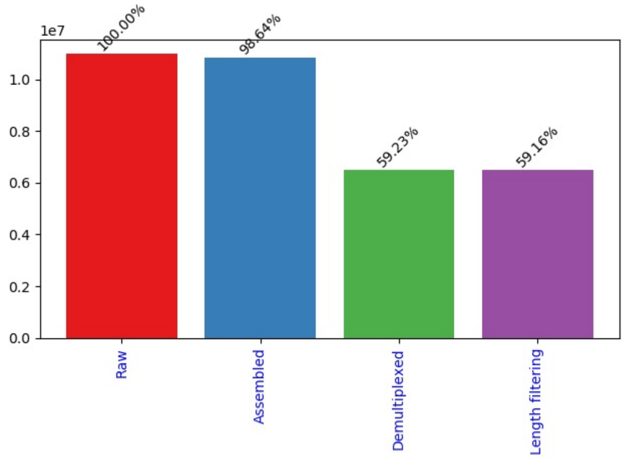

OTU report

Cascabel report on downstream analyses in combination with multiple libraries (if supplied), can be found at the following link: otu_report (CascabelTest/runs/report_test_otu/otu_report_vsearch.html)

References

[FastQC] (1, 2) FastQC v0.11.3. Andrews S. (2010). FastQC: a quality control tool for high throughput sequence data

[PEAR] PEAR: a fast and accurate Illumina Paired-End reAd mergeR. Zhang et al (2014) Bioinformatics 30(5): 614-620 | doi:10.1093/bioinformatics/btt593

[QIIME] (1, 2, 3, 4) QIIME. Caporaso JG, Kuczynski J, Stombaugh J, Bittinger K, Bushman FD, Costello EK, Fierer N, Gonzalez Pena A, Goodrich JK, Gordon JI, Huttley GA, Kelley ST, Knights D, Koenig JE, Ley RE, Lozupone CA, McDonald D, Muegge BD, Pirrung M, Reeder J, Sevinsky JR, Turnbaugh PJ, Walters WA, Widmann J, Yatsunenko T, Zaneveld J, Knight R. 2010. QIIME allows analysis of high-throughput community sequencing data. Nature Methods 7(5): 335-336.

[Cutadapt] Cutadapt v1.15 .Marcel Martin. Cutadapt removes adapter sequences from high-throughput sequencing reads. EMBnet.Journal, 17(1):10-12, May 2011. http://dx.doi.org/10.14806/ej.17.1.200

[Vsearch] Rognes T, Flouri T, Nichols B, Quince C, Mahé F. (2016) VSEARCH: a versatile open source tool for metagenomics. PeerJ 4:e2584. doi: 10.7717/peerj.2584

[uchime] Edgar RC, Haas BJ, Clemente JC, Quince C, Knight R (2011) UCHIME improves sensitivity and speed of chimera detection. Bioinformatics, 27 (16): 2194-2200. doi:10.1093/bioinformatics/btr381.

Author: J. Engelmann & A. Abdala | 2020-03-27
