# Supplementary material for: Cascabel: A Scalable and Versatile Amplicon Sequence Data Analysis Pipeline Delivering Reproducible and Documented Results
Source: Front Genet. 2020 Nov 20;11:489357. doi: 10.3389/fgene.2020.489357 (PMC7718033; doi:10.3389/fgene.2020.489357)
Supplement: Supplementary Datasheet 4—Cascabel OTU report — PDF report describing the OTU analysis of the example 16S sequencing data. It contains the names and locations of all input and output files, names and short description of the modules (“rules”) and parameters which were used in the analysis. In addition, graphics summarize the data in terms of sequence output per sample, number of OTUs and taxonomic composition. [file Data_Sheet_4.PDF]

snakemake report

Amplicon Analysis Report for Libraries: LakeChala

CASCABEL is designed to run amplicon sequence analysis across single or multiple read libraries. This report consists of the OTU creation and taxonomic assignment for all the combined accepted reads of given samples or libraries, if multiple.

User description: Example for single library run with Cascabel using the OTU analysis workflow

Combine Reads

Merge all the reads of the individual libraries into one single file.

Command:

```
cat CascabelTest/runs/report_test/LakeChala_data/seqs_fw_rev_filtered.fasta >
CascabelTest/runs/report_test/seqs_fw_rev_combined.fasta
```

Output file:

- Merged reads: CascabelTest/runs/report_test_otu/seqs_fw_rev_filtered.fasta

The total number of reads is: **6495704**

Benchmark info:

s	max_rss	max_vms	max_uss	max_pss	io_in	io_out	mean_load
6.66	21.36	465.03	13.93	15.19	2737.77	2737.76	0.00

Dereplicate reads

Clusterize the reads with an identity threshold of 100%.

Tool: [vsearch]

Version: **vsearch v2.8.0_linux_x86_64, 754.8GB RAM, 144 cores**

Command:

```
vsearch --derep_fulllength seqs_fw_rev_combined.fasta --output seqs_fw_rev_combined_derep.fasta --uc
seqs_fw_rev_combined_derep.uc --strand both --fasta_width 0 --minuniquesize 1
```

Output files:

- Dereplicated fasta file: CascabelTest/runs/report_test_otu/derep/seqs_fw_rev_combined_derep.fasta

- Cluster file: CascabelTest/runs/report_test_otu/derep/seqs_fw_rev_combined_derep.uc

Total number of dereplicated sequences is: **2783428**

Benchmark info:

s	max_rss	max_vms	max_uss	max_pss	io_in	io_out	mean_load
44.11	2609.00	3122.79	2606.69	2606.79	2737.75	0.00	0.00

Cluster OTUs

Assigns similar sequences to operational taxonomic units, or OTUs, by clustering sequences based on a user-defined similarity threshold.

Tool: [QIIME] - pick_otus.py

Version: **pick_otus.py 1.9.1**

Method: [uclust]

Identity: 0.97

Command:

```
pick_otus.py -m uclust -i CascabelTest/runs/report_test_otu/seqs_fw_rev_filtered.fasta -o
CascabelTest/samples/report_test_otu/otu/ -s 0.97
```

Output files:

- OTU List: CascabelTest/runs/report_test_otu/otu/seqs_fw_rev_filtered_otus.txt

- Log file: CascabelTest/runs/report_test_otu/otu/seqs_fw_rev_filtered_otus.log

The total number of different OTUS is: **254904**

Benchmark info:

s	max_rss	max_vms	max_uss	max_pss	io_in	io_out	mean_load
3238.85	2755.46	7939.77	2751.67	2751.90	830.59	1953.01	0.00

Pick representatives

Pick a single representative sequence for each OTU.

Tool: [QIIME] - pick_rep_set.py

Version: **pick_rep_set.py 1.9.1**

Method: longest

Command:

```
pick_rep_set.py -m longest -i CascabelTest/runs/report_test_otu/otu/seqs_fw_rev_filtered_otus.txt -f
CascabelTest/samples/report_test_otu/seqs_fw_rev_filtered.fasta -o
CascabelTest/samples/report_test_otu/otu/representative_seq_set.fasta --log_fp
CascabelTest/samples/report_test_otu/otu/representative_seq_set.log
```

Output file:

- Fasta file with representative sequences: CascabelTest/runs/report_test_otu/otu/representative_seq_set.fasta

Benchmark info:

s	max_rss	max_vms	max_uss	max_pss	io_in	io_out	mean_load
67.97	2642.25	7502.20	2639.87	2640.09	2399.68	0.02	0.00

Assign taxonomy

Given a set of sequences, assign the taxonomy of each sequence.

Tool: [vsearch]

Version: **vsearch v2.8.0_linux_x86_64, 754.8GB RAM, 144 cores**

Reference fasta file: /export/data01/databases/silva/qiime/SILVA_132_QIIME_release/rep_set/rep_set_all/99/silva132_99.fna

Taxonomy mapping file: /export/data01/databases/silva/qiime/SILVA_132_QIIME_release/taxonomy/taxonomy_all/99/taxonomy_7_levels.txt

Command:

```
vsearch--usearch_global CascabelTest/runs/report_test_otu/otu/representative_seq_set.fasta --db
/export/data01/databases/silva/qiime/SILVA_132_QIIME_release/rep_set/rep_set_all/99/silva132_99.fna --dbmask none --qmask
none --rowlen 0 --id 0.7 --iddef 2 --userfields query+id2+target --maxaccepts 5 --threads 10 --top_hits_only --maxrejects 32 --
output_no_hits --userout representative_seq_set_tax_vsearch.out
```

After taxonomy assignation with vsearch, top hits with the same sequence identity but different taxonomy were mapped to their last common ancestor (LCA) using the script **stampa_merge.py** from https://github.com/frederic-mahe/stampa.

The percentage of successfully assigned OTUs is: **99.99%**

Output file:

- OTU taxonomy assignation: CascabelTest/runs/report_test_otu/otu/taxonomy_vsearch/representative_seq_set_tax_assignments.txt

Benchmark info:

s	max_rss	max_vms	max_uss	max_pss	io_in	io_out	mean_load
351.41	2218.80	3030.86	2216.43	2216.52	673.54	22.66	0.00

Make OTU table

Tabulates the number of times an OTU is found in each sample, and adds the taxonomic predictions for each OTU in the last column.

Tool: [QIIME] - make_otu_table.py

Version: **make_otu_table.py 1.9.1**

Command:

```
make_otu_table.py -i CascabelTest/runs/report_test_otu/otu/taxonomy_vsearch/seqs_fw_rev_filtered_otus.txt -t
CascabelTest/runs/report_test_otu/otu/taxonomy_vsearch/representative_seq_set_tax_assignments.txt -o
CascabelTest/runs/report_test_otu/otu/taxonomy_vsearch/otuTable.biom
```

Output file:

- Biom format table: CascabelTest/runs/report_test_otu/otu/taxonomy_vsearch/otuTable.biom

Benchmark info:

s	max_rss	max_vms	max_uss	max_pss	io_in	io_out	mean_load
21.31	729.94	5602.67	727.30	727.51	165.09	12.33	0.00

Convert OTU table

Convert from the BIOM table format to a human readable format.

Tool: [BIOM]

Version: **biom, version 2.1.6**

Command:

```
biom convert -i CascabelTest/runs/report_test_otu/otu/taxonomy_vsearch/otuTable.biom -o
CascabelTest/runs/report_test_otu/otu/taxonomy_vsearch/otuTable.txt --table-type 'OTU table' --header-key taxonomy --to-tsv
```

Output file:

- TSV format table: CascabelTest/runs/report_test_otu/otu/taxonomy_vsearch/otuTable.txt

Benchmark info:

s	max_rss	max_vms	max_uss	max_pss	io_in	io_out	mean_load
46.68	692.07	5407.96	689.56	689.77	78.89	0.01	0.00

Summarize Taxa

Summarize information of the representation of taxonomic groups within each sample.

Tool: [QIIME] - summarize_taxa.py

Version: **summarize_taxa.py 1.9.1**

Command:

```
summarize_taxa.py -i CascabelTest/runs/report_test_otu/otu/taxonomy_vsearch/otuTable.biom --level 2,3,4,5,6,7 -o
CascabelTest/runs/report_test_otu/otu/taxonomy_vsearch/summary/
```

Output file:

- Taxonomy summarized counts at different taxonomy levels: CascabelTest/runs/report_test_otu/otu/taxonomy_vsearch/summary/otuTable_L**N**.txt

Where **N** is the taxonomy level. Default configuration produces levels from 2 to 6.

Benchmark info:

s	max_rss	max_vms	max_uss	max_pss	io_in	io_out	mean_load
305.12	1110.10	5974.18	1107.68	1107.90	0.00	4.64	0.00

Filter OTU table

Filter OTUs from an OTU table based on their observed counts or identifier.

Tool: [QIIME] - filter_otus_from_otu_table.py

Version: **filter_otus_from_otu_table.py 1.9.1**

Minimum observation counts: 2

Command:

```
filter_otus_from_otu_table.py -i CascabelTest/runs/report_test_otu/otu/taxonomy_vsearch/otuTable.biom -o
CascabelTest/runs/report_test_otu/otu/taxonomy_vsearch/otuTable_noSingletons.biom -n 2
```

Output file:

- Biom table: CascabelTest/runs/report_test_otu/otu/taxonomy_vsearch/otuTable_noSingletons.biom

Benchmark info:

s	max_rss	max_vms	max_uss	max_pss	io_in	io_out	mean_load
25.05	1034.49	5898.41	1031.98	1032.19	78.89	0.02	0.00

Convert Filtered OTU table

Convert the filtered OTU table from the BIOM table format to a human readable format

Tool: [BIOM]

Version: **biom, version 2.1.6**

Command:

```
biom convert -i CascabelTest/runs/report_test_otu/otu/taxonomy_vsearch/otuTable_noSingletons.biom -o
CascabelTest/runs/report_test_otu/otu/taxonomy_vsearch/otuTable_noSingletons.txt --table-type 'OTU table' --header-key
taxonomy --to-tsv
```

Output file:

- TSV format table: CascabelTest/runs/report_test_otu/otu/taxonomy_vsearch/otuTable_noSingletons.txt

Benchmark info:

s	max_rss	max_vms	max_uss	max_pss	io_in	io_out	mean_load
25.05	1034.49	5898.41	1031.98	1032.19	78.89	0.02	0.00

Filter representative sequences

Remove sequences according to the filtered OTU biom table.

Tool: [QIIME] - filter_fasta.py

Version: **filter_fasta.py 1.9.1**

Command:

```
filter_fasta.py -f CascabelTest/samples/report_test_otu/otu/representative_seq_set.fasta -o
CascabelTest/samples/report_test_otu/otu/taxonomy_vsearch/representative_seq_set_noSingletons.fasta -b
CascabelTest/samples/report_test_otu/otu/otuTable_noSingletons.biom
```

Output file:

- Filtered fasta file: CascabelTest/samples/report_test_otu/otu/taxonomy_vsearch/representative_seq_set_noSingletons.fasta

Benchmark info:

s	max_rss	max_vms	max_uss	max_pss	io_in	io_out	mean_load
5.49	329.18	5656.06	326.61	326.82	27.14	21.50	0.00

Align representative sequences

Align the sequences in a FASTA file to each other or to a template sequence alignment.

Tool: [QIIME] - align_seqs.py

Version: TBD

Method: [pynast]

Command:

```
align_seqs.py -m pynast -i CascabelTest/runs/report_test_otu/otu/vsearch/representative_seq_set_noSingletons.fasta -o
CascabelTest/runs/report_test_otu/otu/taxonomy_vsearch/aligned/representative_seq_set_noSingletons_aligned.fasta
```

Output files:

- Aligned fasta file: CascabelTest/runs/report_test_otu/otu/taxonomy_vsearch/aligned/representative_seq_set_noSingletons_aligned.fasta

- Log file: CascabelTest/runs/report_test_otu/otu/taxonomy_vsearch/aligned/representative_seq_set_noSingletons_log.txt

Benchmark info:

s	max_rss	max_vms	max_uss	max_pss	io_in	io_out	mean_load
2269.00	986.56	5872.93	983.77	983.99	95.05	280.18	0.00

Filter alignment

Removes positions which are gaps in every sequence.

Tool: [QIIME] - filter_alignment.py

Version: **filter_alignment.py 1.9.1**

Command:

```
filter_alignment.py -i
CascabelTest/runs/report_test_otu/otu/taxonomy_vsearch/aligned/representative_seq_set_noSingletons_aligned.fasta -o
CascabelTest/runs/report_test_otu/otu/taxonomy_vsearch/aligned/filtered/
```

Output file:

- Aligned fasta file: CascabelTest/runs/report_test_otu/otu/taxonomy_vsearch/aligned/representative_seq_set_noSingletons_aligned_pfiltered.fasta

Benchmark info:

s	max_rss	max_vms	max_uss	max_pss	io_in	io_out	mean_load
416.25	952.86	5812.64	950.23	950.44	628.21	0.02	0.00

Make tree

Create phylogenetic tree (newick format).

Tool: [QIIME] - make_phylogeny.py

Version: **make_phylogeny.py 1.9.1**

Method: [fasttree]

Command:

```
make_phylogeny.py -i
CascabelTest/runs/report_test_otu/otu/taxonomy_vsearch/aligned/representative_seq_set_noSingletons_aligned.fasta -o
representative_seq_set_noSingletons_aligned_pfiltered.tre -t fasttree
```

Output file:

- Taxonomy tree: CascabelTest/runs/report_test_otu/otu/taxonomy_vsearch/aligned/representative_seq_set_noSingletons_aligned.tre

Benchmark info:

s	max_rss	max_vms	max_uss	max_pss	io_in	io_out	mean_load
1691.97	875.80	5852.48	871.29	871.52	39.17	39.00	0.00

Krona report

Krona allows hierarchical data to be explored with zooming, multi-layered pie charts.

Tool: [Krona]

These charts were created using the OTU table **without** singletons

The report was executed for all the samples.

Each sample is represented on a separated chart (same html report).

You can see the report at the following link:

- Krona report: kreport

Or access the html file at:

- Krona html file: CascabelTest/runs/report_test_otu/otu/taxonomy_vsearch/krona_report.html

Benchmark info:

s	max_rss	max_vms	max_uss	max_pss	io_in	io_out	mean_load
35.04	25.78	1019.07	14.85	16.94	47.93	55.09	0.00

Final counts

Following the read counts:

File description	Location	#	(%)
Combined clean reads	CascabelTest/runs/report_test_otu/seqs_fw_rev_combined.fasta	6495704	100%
Dereplicated reads	CascabelTest/runs/report_test_otu/derep/seqs_fw_rev_combined_derep.fasta	2783428	42.85%
OTU table	CascabelTest/runs/report_test_otu/otu/seqs_fw_rev_combined_remapped_otus.txt	254904	3.92%
Taxonomy assignation	CascabelTest/runs/report_test_otu/otu/taxonomy_vsearch/representative_seq_set_tax_assignments.txt	254870	99.99%
OTU table (no singletons: a > 2)	CascabelTest/runs/report_test_otu/otu/taxonomy_vsearch/otuTable_noSingletons.txt	85332	33.48%
Assigned no singletons	CascabelTest/runs/report_test_otu/otu/taxonomy_vsearch/otuTable_noSingletons.txt	85316	99.98%

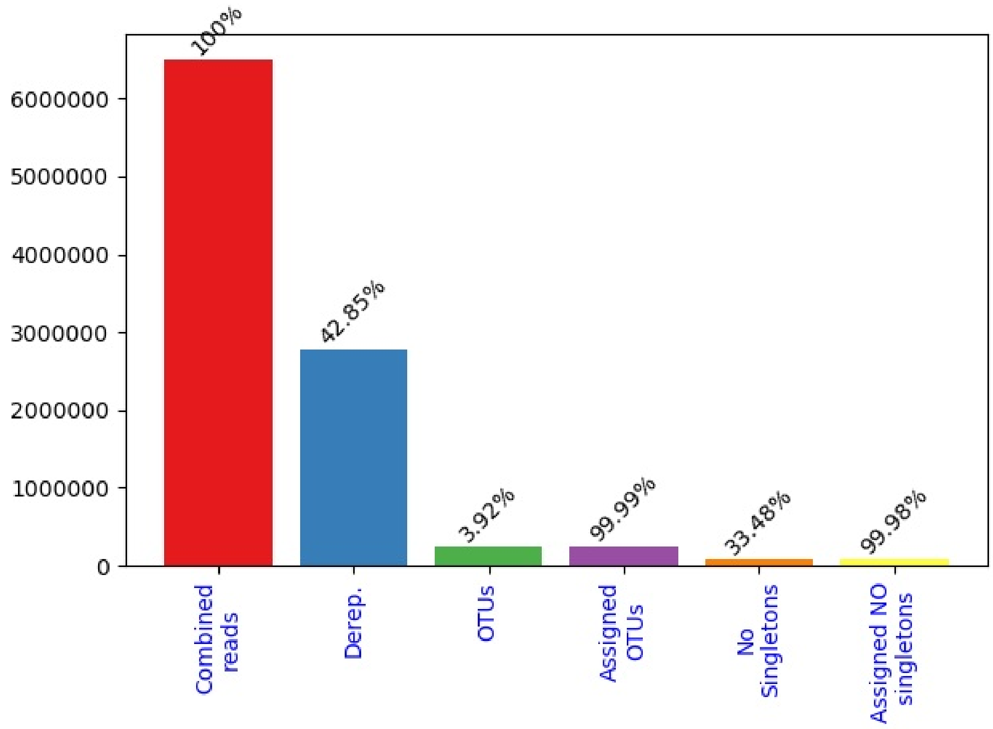

Note:

- Assigned OTUs percentage is the amount of successfully assigned OTUs.

- No singletons percentage is the percentage of no singletons OTUs in reference to the complete OTU table.

- Assigned No singletons is the amount of successfully no singletons assigned OTUs.

References

[QIIME] *(1, 2, 3, 4, 5, 6, 7, 8, 9)* QIIME. Caporaso JG, Kuczynski J, Stombaugh J, Bittinger K, Bushman FD, Costello EK, Fierer N, Gonzalez Pena A, Goodrich JK, Gordon JI, Huttley GA, Kelley ST, Knights D, Koenig JE, Ley RE, Lozupone CA, McDonald D, Muegge BD, Pirrung M, Reeder J, Sevinsky JR, Turnbaugh PJ, Walters WA, Widmann J, Yatsunenko T, Zaneveld J, Knight R. 2010. QIIME allows analysis of high-throughput community sequencing data. Nature Methods 7(5): 335-336.

[Cutadapt] Cutadapt v1.15 .Marcel Martin. Cutadapt removes adapter sequences from high-throughput sequencing reads. EMBnet.Journal, 17(1):10-12, May 2011. http://dx.doi.org/10.14806/ej.17.1.200

[vsearch] *(1, 2)* Rognes T, Flouri T, Nichols B, Quince C, Mahé F. (2016) VSEARCH: a versatile open source tool for metagenomics. PeerJ 4:e2584. doi: 10.7717/peerj.2584

[Krona] Ondov BD, Bergman NH, and Phillippy AM. Interactive metagenomic visualization in a Web browser. BMC Bioinformatics. 2011 Sep 30; 12(1):385.

[BIOM] *(1, 2)* The Biological Observation Matrix (BIOM) format or: how I learned to stop worrying and love the ome-ome. Daniel McDonald, Jose C. Clemente, Justin Kuczynski, Jai Ram Rideout, Jesse Stombaugh, Doug Wendel, Andreas Wilke, Susan Huse, John Hufnagle, Folker Meyer, Rob Knight, and J. Gregory Caporaso.GigaScience 2012, 1:7. doi:10.1186/2047-217X-1-7

[uclust] Edgar RC. 2010. Search and clustering orders of magnitude faster than BLAST. Bioinformatics 26(19):2460-2461.

[pynast] Caporaso JG, Bittinger K, Bushman FD, DeSantis TZ, Andersen GL, Knight R. 2010. PyNAST: a flexible tool for aligning sequences to a template alignment. Bioinformatics 26:266-267.

[fasttree] Price MN, Dehal PS, Arkin AP. 2010. FastTree 2-Approximately Maximum-Likelihood Trees for Large Alignments. Plos One 5(3).

Author: J. Engelmann & A. Abdala | 2020-03-27
