# Supplementary material for: Cascabel: A Scalable and Versatile Amplicon Sequence Data Analysis Pipeline Delivering Reproducible and Documented Results
Source: Front Genet. 2020 Nov 20;11:489357. doi: 10.3389/fgene.2020.489357 (PMC7718033; doi:10.3389/fgene.2020.489357)
Supplement: Supplementary Datasheet 5—Cascabel ASV report — PDF report describing the ASV analysis of the example 16S sequencing data. It contains the names and locations of all input and output files, names and short description of the modules (“rules”) and parameters which were used in the analysis. In addition, the data is summarized in terms of sequence output per sample, number of ASVs and taxonomic composition. [file Data_Sheet_5.PDF]

Amplicon Analysis Report

CASCABEL is designed to run amplicon sequence analysis across single or multiple read libraries. This report consists of the ASV table creation and taxonomic assignment for all the combined accepted reads of given samples or libraries, if multiple.

User description: Example for single library run with Cascabel using the ASV analysis workflow

Filter and Trim

Once that all the individual libraries were demultiplexed, the fastq files from all the samples for all the libraries were processed together.

The filter and trimming steps were both performed with the **filterAndTrim()** function from the R package dada2, according to user parameters.

Tool: dada2

Version: **[1] ‘1.14.1’**

Function: filterAndTrim()

Max Expected Errors (maxEE) FW: 3

Max Expected Errors (maxEE) RV: 5

Forward read truncation: 200

Reverse read truncation: 200

Command:

```
Scripts/asvFilter.R $PWD T 200 200 3 5 10 ",truncQ=2, rm.phix=TRUE" CascabelTest/runs/report_test/asv/filter_summary.out
```

Output file:

- Filtered fastq files: CascabelTest/runs/report_test/<Library>/demultiplexed/filtered/

- Summary: CascabelTest/runs/report_test/asv/filter_summary.out

Note: To speed up downstream computation, consider tightening maxEE. If too few reads are passing the filter, consider relaxing maxEE, perhaps especially on the reverse reads.

Make sure that your forward and reverse reads overlap after length truncation.

Benchmark info:

s	max_rss	max_vms	max_uss	max_pss	io_in	io_out	mean_load
378.85	15403.61	21201.64	13860.07	14040.03	1099.64	971.58	0.00

Amplicon Sequence Variants

In order to identify ASVs, dada2 workflow require to execute several steps. Following a summary of these steps and its main parameters.

Tool: dada2

Version: **[1] ‘1.14.1’**

Learn errors

The first step after filtering the reads is to learn the errors from the fastq files.

Function: learnErrors(filteredFQ)

Error plots:

- FW reads error plot:: CascabelTest/runs/report_test/asv/fw_err.pdf

- RV reads error plot:: CascabelTest/runs/report_test/asv/rv_err.pdf

ASV inference

The amplicon sequence variant identification consists of a high resolution sample inference from the amplicon data using the learned errors.

Function: dada(filteredFQ, errors, pool='pseudo')

Merge pairs

In this step, forward and reverse reads are paired in order to create full denoised sequences.

Function: mergePairs(dadaF, dadaR)

Min overlap: 12

Max mismatch: 0

Length filtering

Sequences that are much longer or shorter than expected may be the result of non-specific priming.

- Shortest length: 242

- Longest length: 262

Remove chimeras

Sequence variants identified as bimeric are removed, and a bimera-free collection of unique sequences is generated.

Function: removeBimeraDenovo()

Method: consensus

Output files:

- Representative ASV sequences: CascabelTest/runs/report_test/asv/representative_seq_set.fasta

The total number of different ASVs is: **2629**

Assign taxonomy

Given a set of sequences, assign the taxonomy of each sequence.

Tool: RDP

Function: assignTaxonomy() *implementation of RDP Classifier within dada2*

Reference database: /export/data01/databases/silva/r132/dada2/silva_nr_v132_train_set.fa.gz

The percentage of successfully assigned ASVs is: **99.70%**

Output file:

- ASV taxonomy assignation: CascabelTest/runs/report_test/asv/taxonomy_dada2/representative_seq_set_tax_assignments.txt

The previous steps were performed within a Cascabel R script according to the following command:

Command

```
Scripts/asvDada2.R $PWD pseudo 10 T selfConsist=FALSE CascabelTest/runs/report_test/asv/ 260 220 10 T
/export/data01/databases/silva/r132/dada2/silva_nr_v132_train_set.fa.gz
/export/data01/databases/silva/r132/dada2/silva_species_assignment_v132.fa.gz T minBoot=45 12 0
```

Benchmark info:

s	max_rss	max_vms	max_uss	max_pss	io_in	io_out	mean_load
8201.23	8875.18	10157.10	8865.45	8868.06	1042.52	1.47	0.00

Make ASV table

Tabulates the number of times an ASV is found in each sample, and adds the taxonomic predictions for each ASV in the last column.

Command:

```
cat CascabelTest/runs/report_test/asv/taxonomy_dada2/representative_seq_set_tax_assignments.txt | awk 'NR==FNR{if(NR>1)
{tax=$2;for(i=3;i<=NF;i++){tax=tax";"$i};h[$1]=tax;}next;} {if(FNR==1){print $0"ttaxonomy"}else{print $0"t"h[$1]}' -
CascabelTest/runs/report_test/asv/asv_table.txt > CascabelTest/runs/report_test/asv/taxonomy_dada2/asvTable.txt
```

Output file:

- ASV table: CascabelTest/runs/report_test/asv/taxonomy_dada2/asvTable.txt

Benchmark info:

s	max_rss	max_vms	max_uss	max_pss	io_in	io_out	mean_load
0.03	•	•	•	•	•	•	0.00

Convert ASV table

Convert from txt to the BIOM table format.

Tool: [BIOM]

Version: **biom, version 2.1.6**

Command:

```
biom convert -i CascabelTest/runs/report_test/asv/taxonomy_dada2/asvTable.txt -o
CascabelTest/runs/report_test/asv/taxonomy_dada2/asvTable.biom --table-type 'OTU table' --table type "OTU table" --to-hdf5 --
process-obs-metdata taxonomy
```

Output file:

- Biom format table: CascabelTest/runs/report_test/asv/taxonomy_dada2/asvTable.biom

Benchmark info:

s	max_rss	max_vms	max_uss	max_pss	io_in	io_out	mean_load
0.91	65.07	4744.77	62.83	62.87	0.00	0.01	0.00

Summarize Taxa

Summarize information of the representation of taxonomic groups within each sample.

Tool: [QIIME] - summarize_taxa.py

Version: **summarize_taxa.py 1.9.1**

Command:

```
summarize_taxa.py -i CascabelTest/runs/report_test/asv/taxonomy_dada2/otuTable.biom --level 2,3,4,5,6,7 -o
CascabelTest/runs/report_test/asv/taxonomy_dada2/summary/
```

Output file:

- Taxonomy summarized counts at different taxonomy levels: CascabelTest/runs/report_test/asv/taxonomy_dada2/summary/otuTable_L**N**.txt

Where **N** is the taxonomy level. Default configuration produces levels from 2 to 6.

Benchmark info:

s	max_rss	max_vms	max_uss	max_pss	io_in	io_out	mean_load
15.78	139.28	5691.64	136.97	137.02	0.81	2.99	0.00

Filter ASV table

Filter ASVs from an ASV table based on their observed counts or identifier.

Tool: [QIIME] - filter_otus_from_otu_table.py

Version: **filter_otus_from_otu_table.py 1.9.1**

Minimum observation counts: 2

Command:

```
filter_otus_from_otu_table.py -i CascabelTest/runs/report_test/asv/taxonomy_dada2/asvTable.biom -o
```

```
CascabelTest/runs/report_test/asv/taxonomy_dada2/asvTable_noSingletons.biom -n 2
```

Output file:

- Biom table: CascabelTest/runs/report_test/asv/taxonomy_dada2/otuTable_noSingletons.biom

Benchmark info:

s	max_rss	max_vms	max_uss	max_pss	io_in	io_out	mean_load
1.87	129.54	5575.93	127.27	127.32	0.82	0.02	0.00

Convert Filtered ASV table

Convert the filtered OTU table from the BIOM table format to a human readable format

Tool: [BIOM]

Version: **biom, version 2.1.6**

Command:

```
biom convert -i CascabelTest/runs/report_test/otu/taxonomy_dada2/asvTable_noSingletons.biom -o
CascabelTest/runs/report_test/asv/taxonomy_dada2/asvTable_noSingletons.txt --table-type 'OTU table' --header-key taxonomy --
to-tsv
```

Output file:

- TSV format table: CascabelTest/runs/report_test/asv/taxonomy_dada2/asvTable_noSingletons.txt

Benchmark info:

s	max_rss	max_vms	max_uss	max_pss	io_in	io_out	mean_load
1.01	72.38	4788.71	70.28	70.32	0.00	0.01	0.00

Filter representative sequences

Remove sequences according to the filtered OTU biom table.

Tool: [QIIME] - filter_fasta.py

Version: **filter_fasta.py 1.9.1**

Command:

```
filter_fasta.py -f CascabelTest/samples/report_test/asv/representative_seq_set.fasta -o
CascabelTest/samples/report_test/asv/taxonomy_dada2/representative_seq_set_noSingletons.fasta -b
CascabelTest/samples/report_test/asv/taxonomy_dada2/otuTable_noSingletons.biom
```

Output file:

- Filtered fasta file: CascabelTest/samples/report_test/asv/taxonomy_dada2/representative_seq_set_noSingletons.fasta

Align representative sequences

Align the sequences in a FASTA file to each other or to a template sequence alignment.

Tool: [QIIME] - align_seqs.py

Version: TBD

Method: [pynast]

Command:

```
align_seqs.py -m pynast -i CascabelTest/runs/report_test/asv/dada2/representative_seq_set_noSingletons.fasta -o
CascabelTest/runs/report_test/asv/taxonomy_dada2/aligned/representative_seq_set_noSingletons_aligned.fasta
```

Output files:

- Aligned fasta file: CascabelTest/runs/report_test/asv/taxonomy_dada2/aligned/representative_seq_set_noSingletons_aligned.fasta

- Log file: CascabelTest/runs/report_test/asv/taxonomy_dada2/aligned/representative_seq_set_noSingletons_log.txt

Benchmark info:

s	max_rss	max_vms	max_uss	max_pss	io_in	io_out	mean_load
70.07	340.83	5686.10	337.20	337.26	51.79	14.61	0.00

Filter alignment

Removes positions which are gaps in every sequence.

Tool: [QIIME] - filter_alignment.py

Version: **filter_alignment.py 1.9.1**

Command:

```
filter_alignment.py -i
CascabelTest/runs/report_test/asv/taxonomy_dada2/aligned/representative_seq_set_noSingletons_aligned.fasta -o
CascabelTest/runs/report_test/asv/taxonomy_dada2/aligned/filtered/
```

Output file:

- Aligned fasta file: CascabelTest/runs/report_test/asv/taxonomy_dada2/aligned/representative_seq_set_noSingletons_aligned_pfiltered.fasta

Benchmark info:

s	max_rss	max_vms	max_uss	max_pss	io_in	io_out	mean_load
12.50	142.64	5668.98	140.34	140.39	19.20	0.96	0.00

Make tree

Create phylogenetic tree (newick format).

Tool: [QIIME] - make_phylogeny.py

Version: **make_phylogeny.py 1.9.1**

Method: [fasttree]

Command:

```
make_phylogeny.py -i
CascabelTest/runs/report_test/asv/taxonomy_dada2/aligned/representative_seq_set_noSingletons_aligned.fasta -o
representative_seq_set_noSingletons_aligned_pfiltered.tre -t fasttree
```

Output file:

- Taxonomy tree: CascabelTest/runs/report_test/asv/taxonomy_dada2/aligned/representative_seq_set_noSingletons_aligned.tre

Benchmark info:

s	max_rss	max_vms	max_uss	max_pss	io_in	io_out	mean_load
18.79	142.04	5694.46	138.03	138.11	1.34	0.96	0.00

Krona report

Krona allows hierarchical data to be explored with zooming, multi-layered pie charts.

Tool: [Krona]

These charts were created using the ASV table **without** singletons

The report was executed for all the samples.

Each sample is represented on a separated chart (same html report).

You can see the report at the following link:

- Krona report: kreport

Or access the html file at:

- Krona html file: CascabelTest/runs/report_test/asv/taxonomy_dada2/krona_report.html

Benchmark info:

s	max_rss	max_vms	max_uss	max_pss	io_in	io_out	mean_load
3.07	34.11	389.77	27.23	28.57	4.70	5.80	0.00

Final counts

Following the read counts:

File description	Location	#	(%)
Demultiplexed reads	CascabelTest/runs/report_test/<LIBRARY>_data/demultiplexed/*.fastq.gz	6484697	100%
QA filtered & trimmed reads	CascabelTest/runs/report_test/<LIBRARY>_data/demultiplexed/filtered/*.fastq.gz	6275118	96.77%
Denoised FW reads	*No intermediate file generated*	6227699	96.04%
Denoised RV reads	*NO intermediate file generated*	6215133	95.84%
Merged and full denoised reads	*No intermediate file generated*	5969708	92.06%
Length filtered	*No intermediate file generated*	5969503	92.06%
Chimera removed	*No intermediate file generated*	5375705	82.90%
ASV table	CascabelTest/runs/report_test/asv/asvTable.txt	2629	0.04%
Taxonomy assignation	CascabelTest/runs/report_test/asv/taxonomy_dada2/representative_seq_set_tax_assignments.txt	2621	99.70%
ASV table (no singletons: a > 2)	CascabelTest/runs/report_test/asv/taxonomy_dada2/asvTable_noSingletons.txt	2626	99.89%
Assigned no singletons	CascabelTest/runs/report_test/otu/taxonomy_vsearch/asvTable_noSingletons.txt	2618	99.70%

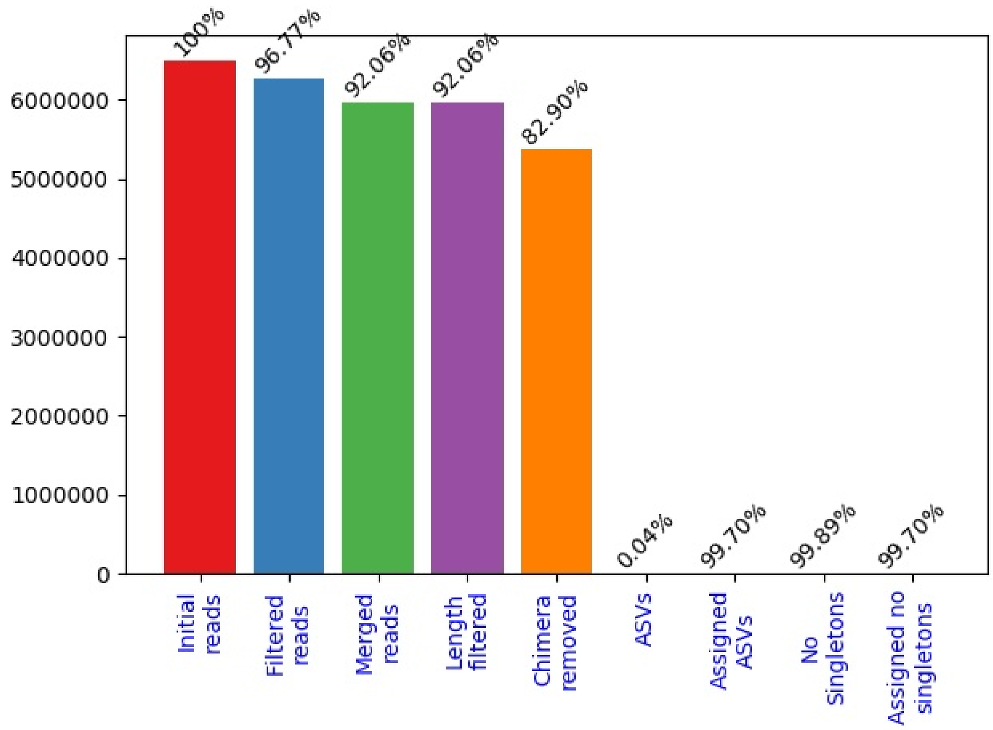

Note:

- Assigned ASVs percentage is the amount of successfully assigned ASVs.

- No singletons percentage is the percentage of no singletons ASVs in reference to the complete ASV table.

- Assigned No singletons is the amount of successfully no singletons assigned ASVs.

References

[QIIME] *(1, 2, 3, 4, 5, 6)* QIIME. Caporaso JG, Kuczynski J, Stombaugh J, Bittinger K, Bushman FD, Costello EK, Fierer N, Gonzalez Pena A, Goodrich JK, Gordon JI, Huttley GA, Kelley ST, Knights D, Koenig JE, Ley RE, Lozupone CA, McDonald D, Muegge BD, Pirrung M, Reeder J, Sevinsky JR, Turnbaugh PJ, Walters WA, Widmann J, Yatsunenko T, Zaneveld J, Knight R. 2010. QIIME allows analysis of high-throughput community sequencing data. Nature Methods 7(5): 335-336.

[Cutadapt] Cutadapt v1.15 .Marcel Martin. Cutadapt removes adapter sequences from high-throughput sequencing reads. EMBnet.Journal, 17(1):10-12, May 2011. http://dx.doi.org/10.14806/ej.17.1.200

[vsearch] Rognes T, Flouri T, Nichols B, Quince C, Mahé F. (2016) VSEARCH: a versatile open source tool for metagenomics. PeerJ 4:e2584. doi: 10.7717/peerj.2584

[Krona] Ondov BD, Bergman NH, and Phillippy AM. Interactive metagenomic visualization in a Web browser. BMC Bioinformatics. 2011 Sep 30; 12(1):385.

[BIOM] *(1, 2)* The Biological Observation Matrix (BIOM) format or: how I learned to stop worrying and love the ome-ome. Daniel McDonald, Jose C. Clemente, Justin Kuczynski, Jai Ram Rideout, Jesse Stombaugh, Doug Wendel, Andreas Wilke, Susan Huse, John Hufnagle, Folker Meyer, Rob Knight, and J. Gregory Caporaso.GigaScience 2012, 1:7. doi:10.1186/2047-217X-1-7

[RDP] Wang, Q, G. M. Garrity, J. M. Tiedje, and J. R. Cole. 2007. Naive Bayesian Classifier for Rapid Assignment of rRNA Sequences into the New Bacterial Taxonomy. Appl Environ Microbiol. 73(16):5261-7.

[dada2] Callahan BJ, McMurdie PJ, Rosen MJ, Han AW, Johnson AJA, Holmes SP (2016). DADA2: High-resolution sample inference from Illumina amplicon data. Nature Methods, 13, 581-583. doi: 10.1038/nmeth.3869.

[pynast] Caporaso JG, Bittinger K, Bushman FD, DeSantis TZ, Andersen GL, Knight R. 2010. PyNAST: a flexible tool for aligning sequences to a template alignment. Bioinformatics 26:266-267.

[fasttree] Price MN, Dehal PS, Arkin AP. 2010. FastTree 2-Approximately Maximum-Likelihood Trees for Large Alignments. Plos One 5(3).

Author: J. Engelmann & A. Abdala | 2020-06-18
