# Supplementary material for: Cascabel: A Scalable and Versatile Amplicon Sequence Data Analysis Pipeline Delivering Reproducible and Documented Results
Source: Front Genet. 2020 Nov 20;11:489357. doi: 10.3389/fgene.2020.489357 (PMC7718033; doi:10.3389/fgene.2020.489357)
Supplement: Supplementary Datasheet 6—Folder structure of results of an OTU analysis — This folder structure is generated for the output of an OTU analysis performed with Cascabel. Note that only the main files are displayed, log files and temporary files are not shown. [file Data_Sheet_6.PDF]

File structure for generating OTUs (main files):

```
├── metadata
│   ├── bc_validation
│   │   └── <LIB_NAME>
│   │       └── sampleList_mergedBarcodes_<LIB_NAME>.html   // barcode HTML report
│   └── sampleList_mergedBarcodes_<LIB_NAME>.txt            // /path/to/original/bacode/file.tsv
├── runs
│   └── <RUN_NAME>                               //results for this RUN
│       ├── <LIB_NAME>_data                      //processed data per library: <LIB_NAME>
│       │   ├── barcodes                         //extracted barcodes
│       │   ├── barcodes_unassigned              //extracted barcodes (reverse complement unassigned reads)
│       │   ├── chimera                          //chimera sequences
│       │   ├── demultiplexed                    //demultiplexed fastq files
│       │   ├── peared
│       │   │   ├── seqs.assembled.fastq         //forward-reverse assembled reads
│       │   │   └── qc                           //QC on assembled reads
│       │   ├── splitLibs                        //split library based on forward matching barcodes
│       │   ├── splitLibsRC                      //split library based on reverse complemented barcodes
│       │   ├── seqs_fw_rev_accepted.fna         //fasta file with demultiplexed reads
│       │   └── seqs_fw_rev_filtered.fasta       //demultiplexed fasta file after length filtering
│       ├── otu                                  //OTU analysis (ALL LIBRARIES)
│       │   ├── representative_seq_set.fasta     //fasta file with one representative sequence per OTU
│       │   └── taxonomy_<TOOL>      //directory with the taxonomy assignation by <tool>: vsearch|blast|qiime
│       │       ├── aligned                      //directory with the phylogenetics data
│       │       │   ├── filtered                 //aligment with the p_filtered singleton reads
│       │       │   │   ├── representative_seq_set_noSingletons_aligned_pfiltered.fasta //sequence alignment
│       │       │   │   └── representative_seq_set_noSingletons_aligned_pfiltered.tre   //phylogenetic tree
│       │       │   └── representative_seq_set_noSingletons_aligned.fasta //alignment with singletons
│       │       ├── otuTable.biom                              //biom table
│       │       ├── otuTable_noSingletons.biom                 //biom table without singletons
│       │       ├── otuTable_noSingletons.txt                  //biom table (txt format) without singletons
│       │       ├── otuTable.txt                               //biom table (txt format)
│       │       ├── representative_seq_set_noSingletons.fasta   //fasta file with one representative sequence
│       │       │                                              // per OTU without singletons
│       │       ├── representative_seq_set_tax_<TOOL>.out   //output file with taxonomy assignments
│       │       │                                          //<tool>: vsearch|blast|qiime
│       │       └── summary                      //directory with OTU tables in biom and txt format,
│       │                                        // summarized at different taxonomic levels.
│       ├── otu_report_<TOOL>.html      //OTU report for ALL libraries, taxonomy <tool>: vsearch|blast|qiime
│       ├── otu_report_<TOOL>.pdf                //same as previous in pdf format
│       ├── report_<LIB_NAME>_<TOOL>.html        //individual report for each library (<LIB_NAME>)
│       ├── report_<LIB_NAME>_<TOOL>.pdf         //same as previous in pdf format
│       ├── report_<TOOL>.zip                    //zip file with html reports and their resources -
│       │                                        // "portable report"
│       ├── report_files                         //directory with all resources for the html reports
│       │   └── krona_report.<TOOL>.html         //KRONA report for all samples
│       └── seqs_fw_rev_combined.fasta           //concatenated filtered fasta files per library
└── samples                                      //directory with raw sequence data and QC reports
    └── <LIB_NAME>                               //one directory per sequencing library
        ├── qc                                   //quality control files
        │   ├── fw_fastqc.html                   //Fastqc report for forward reads
        │   └── rv_fastqc.html                   //Fastqc report for reverse reads
        └── rawdata
            ├── fw.fastq -> /path/to/raw/fw-reads.fq   //original (raw) fastq files, forward reads
            └── rv.fastq -> /path/to/raw/rv-reads.fq   //original (raw) fastq files, reverse reads
```
