# Supplementary material for: Cascabel: A Scalable and Versatile Amplicon Sequence Data Analysis Pipeline Delivering Reproducible and Documented Results
Source: Front Genet. 2020 Nov 20;11:489357. doi: 10.3389/fgene.2020.489357 (PMC7718033; doi:10.3389/fgene.2020.489357)
Supplement: Supplementary Datasheet 7—Folder structure of results of an ASV analysis — This folder structure is generated for the output of an ASV analysis performed with Cascabel. Note that only the main files are displayed, log files and temporary files are not shown. [file Data_Sheet_7.PDF]

File structure for generating ASVs (main files):

```
├── metadata
│   ├── bc_validation
│   │   └── <LIB_NAME>
│   │       └── sampleList_mergedBarcodes_<LIB_NAME>.html   // barcode HTML report
│   └── sampleList_mergedBarcodes_<LIB_NAME>.txt            // /path/to/original/bacode/file.tsv
├── runs
│   └── <RUN_NAME>                                  //results for this RUN
│       ├── <LIB_NAME>_data                         //processed data per library: <LIB_NAME>
│       │   ├── barcodes                            //extracted barcodes
│       │   ├── barcodes_unassigned                 //extracted barcodes (reverse complement unassigned reads)
│       │   ├── chimera                             //chimera sequences
│       │   ├── demultiplexed                       //demultiplexed fastq files
│       │   ├── peared
│       │   │  ├── seqs.assembled.fastq             //forward-reverse assembled reads
│       │   │  └── qc                               //QC on assembled reads
│       │   ├── splitLibs                           //split library based on forward matching barcodes
│       │   ├── splitLibsRC                         //split library based on reverse complemented barcodes
│       │   ├── seqs_fw_rev_accepted.fna            //fasta file with demultiplexed reads
│       │   └── seqs_fw_rev_filtered.fasta          //demultiplexed fasta file after length filtering
│       ├── asv                                     //ASV analysis (ALL LIBRARIES)
│       │   ├── filter_summary.out                  //summary of dada2 sequence filtering
│       │   ├── representative_seq_set.fasta        //ASV representative sequences
│       │   └── taxonomy_<TOOL>                     //taxonomy assignment of the ASV sequences
│       │       ├── aligned                         //directory with the phylogenetics data
│       │       │   ├── filtered                    //aligment with the p_filtered singleton reads
│       │       │   │   ├── representative_seq_set_noSingletons_aligned_pfiltered.fasta //sequence alignment
│       │       │   │   └── representative_seq_set_noSingletons_aligned_pfiltered.tre   //phylogenetic tree
│       │       │   └── representative_seq_set_noSingletons_aligned.fasta //alignment with singletons
│       │       ├── asvTable.biom                   //ASV biom table
│       │       ├── asvTable_noSingletons.biom      //ASV biom table without singletons
│       │       ├── asvTable_noSingletons.txt       //ASV biom table without singletons in text format
│       │       ├── asvTable.txt                    //ASV biom table in text format
│       │       ├── representative_seq_set_tax_assignments.txt //fasta file with ASV sequences and taxonomy
│       │       │                                              // assignments
│       │       └── summary                       //directory with ASV tables in biom and txt format,
│       │                                         //summarized at different taxonomic levels.
│       ├── asv_report_<TOOL>.html                //ASV report for ALL libraries
│       ├── asv_report_<TOOL>.pdf                 //same as previous in pdf format
│       ├── report_<LIB_NAME>_<TOOL>.html         //individual report for each library (<LIB_NAME>)
│       ├── report_<LIB_NAME>_<TOOL>.pdf          //same as previous in pdf format
│       ├── report_<TOOL>.zip                      //zip file with html reports and their resources -
│       │                                         // "portable report"
│       ├── report_files                          //directory with all resources for the html reports
│       │   └── krona_report.<TOOL>.html          //KRONA report for all samples
│       └── seqs_fw_rev_combined.fasta            //concatenated filtered fasta files per library
└── samples                                       //directory with raw sequence data and QC reports
    └── <LIB_NAME>                                //one directory per sequencing library
        ├── qc                                    //quality control files
        │   ├── fw_fastqc.html                    //Fastqc report for forward reads
        │   └── rv_fastqc.html                    //Fastqc report for reverse reads
        └── rawdata
            ├── fw.fastq -> /path/to/raw/fw-reads.fq   //original (raw) fastq files, forward reads
            └── rv.fastq -> /path/to/raw/rv-reads.fq   //original (raw) fastq files, reverse reads
```
